# Structure-Guided Computational Methods Predict Multiple Distinct Binding Modes for Pyrazoloquinolinones in GABA_A_ Receptors

**DOI:** 10.3389/fnins.2020.611953

**Published:** 2021-01-15

**Authors:** Jure Fabjan, Filip Koniuszewski, Benjamin Schaar, Margot Ernst

**Affiliations:** Department of Pathobiology of the Nervous System, Center for Brain Research, Medical University of Vienna, Vienna, Austria

**Keywords:** GABA_A_ receptor, allosteric modulation, pyrazoloquinolinone, functional selectivity, computational docking, pharmacophore analysis

## Abstract

Pyrazoloquinolinones (PQs) are a versatile class of GABA_A_ receptor ligands. It has been demonstrated that high functional selectivity for certain receptor subtypes can be obtained by specific substitution patterns, but so far, no clear SAR rules emerge from the studies. As is the case for many GABA_A_ receptor targeting chemotypes, PQs can interact with distinct binding sites on a given receptor pentamer. In pentamers of αβγ composition, such as the most abundant α1β2γ2 subtype, many PQs are high affinity binders of the benzodiazepine binding site at the extracellular α+/γ2− interfaces. There they display a functionally near silent, flumazenil-like allosteric activity. More recently, interactions with extracellular α+/β− interfaces have been investigated, where strong positive modulation can be steered toward interesting subtype preferences. The most prominent examples are functionally α6-selective PQs. Similar to benzodiazepines, PQs also seem to interact with sites in the transmembrane domain, mainly the sites used by etomidate and barbiturates. This promiscuity leads to potential contributions from multiple sites to net modulation. Developing ligands that interact exclusively with the extracellular α+/β− interfaces would be desired. Correlating functional profiles with binding sites usage is hampered by scarce and heterogeneous experimental data, as shown in our meta-analysis of aggregated published data. In the absence of experimental structures, bound states can be predicted with pharmacophore matching methods and with computational docking. We thus performed pharmacophore matching studies for the unwanted sites, and computational docking for the extracellular α1,6+/β3− interfaces. The results suggest that PQs interact with their binding sites with diverse binding modes. As such, rational design of improved ligands needs to take a complex structure-activity landscape with branches between sub-series of derivatives into account. We present a workflow, which is suitable to identify and explore potential branching points on the structure-activity landscape of any small molecule chemotype.

## Introduction

The WHO Model List of Essential Medicines contains many allosteric modulators of GABA_A_ receptors, among them several benzodiazepines and many sedative general anesthetics such as propofol. Despite their big usefulness, side effects are associated with all of them. One of the promising avenues to produce improved GABA_A_ receptor targeting medications is the exploitation of subtype selective targeting (Olsen and Sieghart, 2009; Sieghart and Savic, 2018). In this vein, functionally selective ligands, i.e., ligands which exert allosteric effects at certain subtypes while binding “silently” to other subtypes, have gained considerable attention over the last years (Rudolph and Knoflach, 2011; Skolnick, 2012; Sieghart and Savic, 2018). The most advanced functionally selective compound so far seems to be basmisanil, a functionally α5-selective negative modulator acting at the benzodiazepine binding site (Liogier d’Ardhuy et al., 2015; Roche, 2016).

In addition to compounds, which target the high affinity benzodiazepine binding sites, functionally selective ligands have been described for other binding sites such as the site at which the general anesthetic etomidate binds (Sieghart and Savic, 2018), a modulatory site at extracellular α+/β− interfaces (Varagic et al., 2013a), and for some functionally selective ligands the binding sites have not been identified (Sieghart and Savic, 2018). The extracellular interfaces show greater sequence diversity compared to the binding site in the transmembrane domain (TMD) (Puthenkalam et al., 2016), and thus are considered highly promising targets to obtain compounds with a narrow subtype preference profile.

Since the first description of the ECD (extracellular domain) α+/β− as a modulatory site for pyrazoloquinolinones (PQs), considerable effort was invested to explore the potential for subtype selective targeting of this site (Ramerstorfer et al., 2011; Varagic et al., 2013a, b; Mirheydari et al., 2014; Simeone et al., 2017, 2019; Knutson et al., 2018; Treven et al., 2018). Recently this site has been claimed to mediate the anxiolytic effects of etifoxine (Mattei et al., 2019) and is considered as a promising target for drugs devoid of some of the side effects displayed by the popular benzodiazepine targeting medications, such as benzodiazepines themselves (diazepam, alprazolam, etc.) or the Z-drugs (zolpidem, zopiclone, and zaleplon). PQs comprise the ECD α+/β− targeting scaffold with the highest number of ligands which have been studied, and have delivered prototypical functionally a6-preferring compounds (Varagic et al., 2013a; Knutson et al., 2018; Treven et al., 2018; Simeone et al., 2019) and compounds with affinity and efficacy β1-selective profiles (Simeone et al., 2017). Yet little is known about the precise molecular determinants of their interactions with GABA_A_ receptors.

**FIGURE 1:**
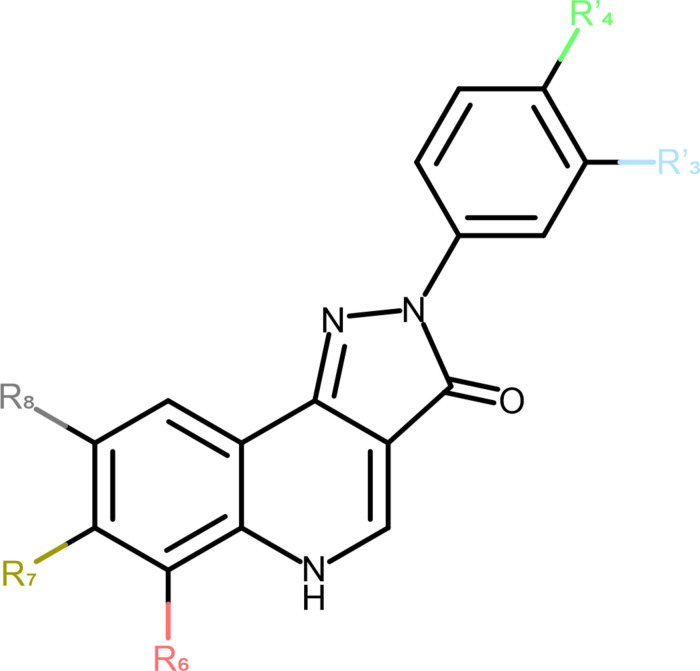
Schematic representation of the PQ scaffold. The positions of substitutions are marked to match Table 1 on the 2D structure.

Figure 1 pyrazoloquinolinones were first introduced as high affinity ligands of the benzodiazepine binding site (Zhang et al., 1995; Savini et al., 1998, 2001; Carotti et al., 2003). Many years after their introduction it was realized that CGS 9895 does not elicit any modulatory effect by its interaction with the benzodiazepine binding site, but rather modulates GABA elicited currents by the so-called modulatory PQ (mPQ) site at the ECD α+/β− interface (Ramerstorfer et al., 2011; Sieghart et al., 2012). The first subtype selective PQs with pronounced functional selectivity for α6β2,3γ2 receptors over all other α isoforms were presented in 2013, and the description of compounds with β1-preferring profiles followed in 2017 (Varagic et al., 2013a; Simeone et al., 2017). The involvement of the ECD α+/β− interface was demonstrated in three separate studies (Ramerstorfer et al., 2011; Varagic et al., 2013b; Maldifassi et al., 2016), of which one postulated additional binding sites that overlap with the “low affinity diazepam sites” (Walters et al., 2000; Maldifassi et al., 2016). Thus, for a rational improvement of selectivity profiles more insight is needed concerning the use of multiple binding sites in a given receptor pentamer (Iorio et al., 2020).

Further improvement of PQs seems very promising as several members of this scaffold have already demonstrated low toxicity and considerable clinical promise in the seventies and eighties. After the introduction of the functional selectivity potential, pre-clinical studies demonstrated promise for novel indications such as sensorimotor gating deficits (Chiou et al., 2018), trigeminal neuropathic pain (Puri et al., 2012; Vasovic et al., 2019) and migraine (Fan et al., 2018).

Thus, a detailed understanding (ideally at the atom level) of their mode of action and the molecular determinants of selective interactions is highly desired to accelerate development of PQs that might be suitable as drugs (Knutson et al., 2018). Here we present a thorough re-analysis of the experimental data since 2011 (Ramerstorfer et al., 2011; Varagic et al., 2013a, b; Mirheydari et al., 2014; Maldifassi et al., 2016; Simeone et al., 2017, 2019; Treven et al., 2018) and complement it with a computational analysis of functionally selective and unselective PQs to shed light on the molecular determinants of their complex pharmacological profile.

## Materials and Methods

### Aggregation of Relevant Data From the Literature

From the selected papers we extracted efficacies for each compound (Table 1) and receptor combination (including the mutated receptors) (Ramerstorfer et al., 2011; Varagic et al., 2013a, b; Mirheydari et al., 2014; Maldifassi et al., 2016; Simeone et al., 2017, 2019; Treven et al., 2018). Tabulated efficacy was extracted for the concentration, at which the modulation was the highest, except in the case of one study (Simeone et al., 2017), where calculated maximal efficacies were taken from the Supplementary Information. Where the mean modulation per compound concentration was given in the Supplementary, we used the data from such table. Else, the efficacy was estimated from the graphs. For each extracted value we also noted if the value represents the maximal efficacy based on the graphs. The whole dataset is available in Supplementary Table 1.

**TABLE 1:**
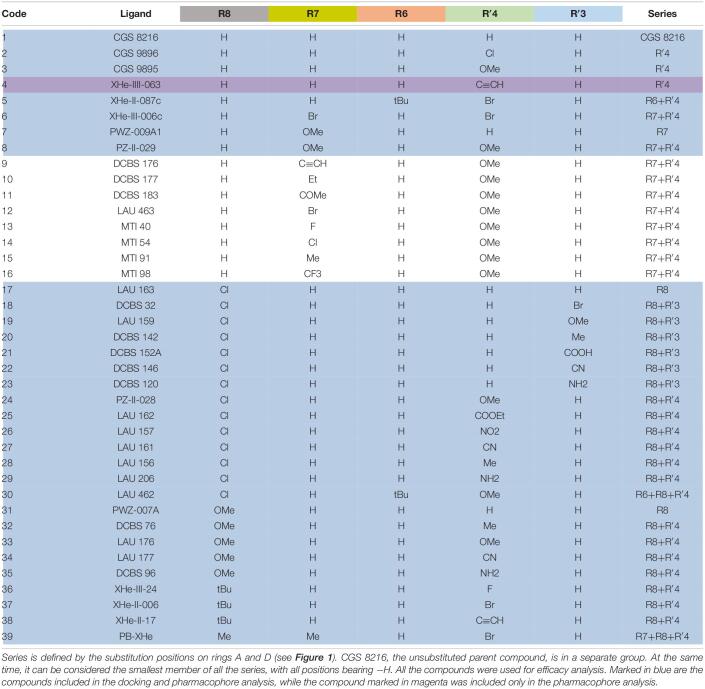
List of compounds from the selected papers, together with their substitution pattern.

*Series is defined by the substitution positions on rings A and D (see Figure 1). CGS 8216, the unsubstituted parent compound, is in a separate group. At the same time, it can be considered the smallest member of all the series, with all positions bearing −H. All the compounds were used for efficacy analysis. Marked in blue are the compounds included in the docking and pharmacophore analysis, while the compound marked in magenta was included only in the pharmacophore analysis.*

### Ligand Preparation

The ligand structures were prepared and energy minimized in MOE 2019.0102 (Molecular Operating Environment [MOE], 2020), and saved in mol2 format. For pharmacophore analysis with LigandScout the SMILES code of each compound was extracted.

### Structure-Based Pharmacophore Analysis

Structure-based 3D pharmacophore screening was performed with MOE and LigandScout 4.4.4 (Wolber and Langer, 2005). For LigandScout the “match all query features” screening mode was used with the Pharmacophore-Fit scoring function. In MOE the unified pharmacophore algorithm was used. For the 3D structure-based pharmacophore modeling the cryo-electron microscopy-derived structures of GABA_A_ receptors were used (PDB IDs: 6HUP, 6HUO, 6HUK, 6D6T, 6D6U, and 6X3V (Zhu et al., 2018; Masiulis et al., 2019; Kim et al., 2020); 6X3V was published after this study was completed, and was utilized for a smaller number of screens). Two different pharmacophore screening series were performed. In the first series (screens 1.1, 1.2, see Supplementary Table 2) both programs were used. The pharmacophore features of each structure were automatically generated by each program, and default settings were used together with the option to omit features in the matching step. Table 2 lists the range of settings, Supplementary Figure 1 shows representative features, and Supplementary Table 2 provides the specific settings.

**TABLE 2:** Range of settings used in the pharmacophore screens.

	LigandScout	MOE
Identified features*	3–8*	14–21*
Omitted features	1–5	8–14
Exclusion spheres	Off or Default	None or 1.8 Å
Ligand shape radius	n.a.	None or 2–3.5 Å

See Supplementary Tables 2–6 for the parameters used in the 13 screens. *The number of identified features depends on the input PDB, and on the thresholds unique for each program.

In the second series of screens with MOE, program features were explored for the best replication of known binders to the TMD β+/α− (see Table 2, results, and Supplementary Tables 3–6).

### Protein Preparation

For ECD α6+/β3− binding site we created an α6β3γ2 model with Modeler 9.23 (Webb and Sali, 2016). The used template was 6HUP. As the substitution of the α subunit does not result in any INDELs in the ECD and TMD, (Supplementary Figure 2) standard settings without alignment optimization were applied.

The docking was performed for ECD α1+/β3− and ECD α6+/β3− binding sites. The ECD α1+/β3− binding site structure was taken from 6HUP (Masiulis et al., 2019). GABA was transferred into the binding site of interest by superposing it to ECD β3+/α1−. Then ECD α1+/β3− subunits and the ligand were saved as mol2 file. In the same manner as for the ECD α1+/β3− pocket, we transferred GABA from 6HUP ECD β3+/α1− to the appropriate pocket of the α6β3γ2 model. The two chains of interest and the ligand were then saved as mol2 file.

### Computational Docking

For docking we used Gold v.2020.1 (Jones et al., 1997). The template configuration file was generated using Hermes GUI and then used through CSD Python API by substituting the docked ligand.

Proteins were first prepared by adding hydrogens and extracting the template ligands from the pockets. Binding sites were defined as a volume 7 Å from the template ligand. We set autoscale on 2, number of generated poses on 200, and disabled early termination. Generated poses were evaluated with chemscore. For bigger amino acids around the template ligand the rotamer library was set on free and for four amino acids on loop C (α1Ser205, α1Ser206, α1Thr207, and α1Gly208) soft potentials were used. The remaining settings were left on default.

### Analysis of Docking Results

We extracted scoring and rescoring results from the docking runs using CSD Python API. Furthermore, the ligand-protein complexes were generated for each pose and saved as PDB files. By using MOE, we measured spherical coordinates of a subset of core ligand atoms for each pose complex (Supplementary Figure 3). The coordinate system was defined by Cα-atoms of pre-defined amino acids–α1Gly208 as origin and α1Ser205, α1Tyr210, and β3Met115 as axes, with homologous amino acids used for ECD α6+/β3−.

The coordinates were analyzed in R v4.0.2 (R Core Team, 2020) using an adapted “phenotypic earth mover’s distance” pipeline, described by Chen et al. (2020). RMSD of the compound atoms was used as distance between samples in hierarchical clustering. The number of clusters used for ECD α1+/β3− and ECD α6+/β3− binding sites were 80 and 40, respectively. Subsequently, the clusters in which no compound had more than 10 poses were discarded with the poses not considered in the following analysis. After the removal, the ECD α1+/β3− docking results retained 4979, while the ECD α6+/β3− docking results retained 5755 out of 6000 poses. In the next step every compound was described as a distribution of the sums of chemscores for all poses in a cluster. To calculate the difference between individual clusters, principal component analysis was first used to reduce the dimensionality of the circular coordinates. Coordinates of connected atoms have a high level of cross-correlation (Supplementary Figure 4). As principal components are orthogonal to each other, after the PCA step cross-correlation is eliminated. Subsequently, the first three components were used to calculate the distances between cluster centroids as a measure of their dissimilarity. The chemscore distributions and the distances between cluster centroids were then used to compute ligand dissimilarity with earth mover’s distance. The resulting distance matrix was used to compute a 3D diffusion map of the ligands.

## Results

### Ligand Properties in Functional Studies

Efficacy data was aggregated from all functional PQ studies since the seminal report of the ECD α1+/β3− binding site (Ramerstorfer et al., 2011; Varagic et al., 2013a, b; Mirheydari et al., 2014; Maldifassi et al., 2016; Simeone et al., 2017, 2019; Treven et al., 2018). Due to the different compound solubilities and apparent potencies, in many cases full dose response curves could not be obtained, and thus maximum efficacy can be extrapolated only from some datasets. To be able to compare efficacies across the compounds, the modulation elicited at 10 μM compound concentration was used whenever available. It should be noted that this data might not only reflect differences in the theoretical maximum efficacy, but also the diverse positions of the data points in the respective dose response curves i.e., close to maximal efficacy, rising phase, or in the case of biphasic responses rising or falling phase (Supplementary Table 1). However, the gross trends in efficacy remain valid and interesting to derive potential structure-activity relations and to correlate structural hypotheses with the experimental data.

**FIGURE 2:**
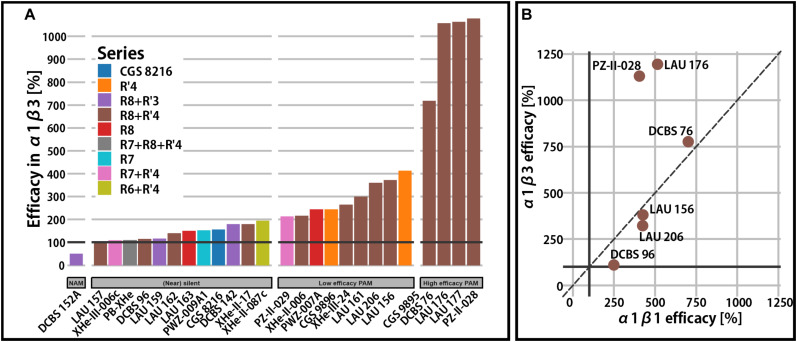
Efficacy in binary α1β receptors. **(A)** Qualitative overview of total modulatory efficacy (% change of the reference GABA current at 100%) observed in α1β3 receptors at a condition used most in the experimental studies. Bars are colored by the compound series. A black horizontal line is placed on 100% to delineate the point of no modulatory effect. **(B)** Comparison of calculated maximal efficacy between α1β3 and α1β1 for six compounds from the R8+R′4 series.

Functional data for most of the compounds in Table 1 exists in α1β3 receptors as this was the receptor isoform that was used to demonstrate the existence of the modulatory ECD α1+/β3− binding site of PQs (Ramerstorfer et al., 2011; Figure 2A).

For a broad range of compounds and substitution patterns, we observe (near) silent interactions with the α1β3 receptors (Figure 2A). Silent or near silent binding in α1β3 receptors renders ligands with this property potential candidates for selective agents in non-α1 or non-β3 assemblies. While many compounds are known to elicit higher efficacy in β2- and β3-containing receptors, very few functionally β1-selective compounds have been described so far (Simeone et al., 2017). In a small study involving six PQs, amino-substituted compounds have been demonstrated to display functional β1-preference (Figure 2B). Specifically, DCBS 96 exhibits functional selectivity, as it is a silent modulator in non-β1 receptors (Simeone et al., 2017), while LAU 206 displays a slight functional β1-preference.

**FIGURE 3:**
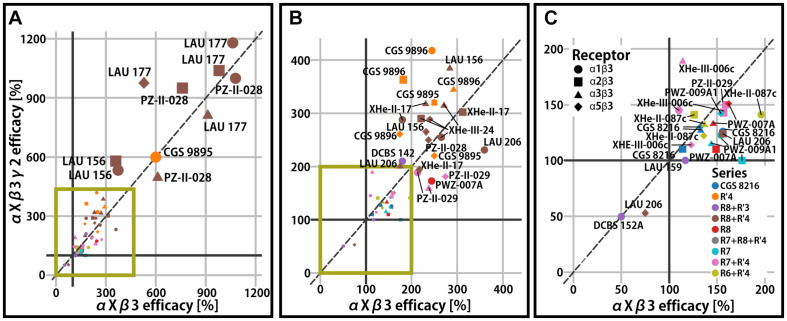
Efficacy of γ2-containing receptors compared to the respective αxβ3 (*x* = 1, 2, 3, 5) reference receptor. **(A)** All compounds displayed on the full efficacy scale. Efficacy is the % change of the reference GABA current at 100%. High efficacy compounds are labeled. The green box indicates which part of the graph is enlarged in panels **(B,C)**. **(B)** The scale is chosen to accommodate mid-efficacy compounds. **(C)** The scale is chosen for low efficacy and negative modulator compounds. Legends for the color code (ligand series) and symbol use (α isoform) for all panels are displayed.

Binary αβ receptors have been confirmed to exist, but are thought to represent a small population of native receptors (Olsen and Sieghart, 2009; Mortensen et al., 2011), while the majority of receptors in the mammalian CNS contain a γ2 subunit. The γ2 subunit confers benzodiazepine sensitivity and high affinity PQ binding to receptors (Sieghart and Savic, 2018). The impact of the γ2 subunit on PQ efficacy has been demonstrated to be relatively low (Ramerstorfer et al., 2011; Varagic et al., 2013a, b), though there are a few cases with modest impact (see Figure 3A). For example, PWZ-009A1 positively modulates α2β3 receptors (176%), but not α2β3γ2 (Figure 3C). On the other hand, the introduction of γ2 potentiates the efficacy of CGS 9896 by two-fold (from 182% in binary to 363%) in α2β3-containing receptors (Figure 3B). Thus, even though the integration of the γ2 subunit in the receptors is incomplete and variable in oocytes (Baburin et al., 2008), a clear influence is seen for specific combinations of compound and α-isoform. This might be due to allosteric coupling of interactions between the ECD αx+/γ2− and ECD αx+/β3− site. Low efficacy modulation in the nM range by interactions with specific ECD αx+/γ2− interfaces has been probed with concatenated subunits (Simeone et al., 2019), as well as with the use of steric hindrance/cysteine mutations in position γ2M130 (Ramerstorfer et al., 2011; Varagic et al., 2013a, b). It has been concluded that the high affinity binding at this site can, for some compounds and in some αx+/γ2− sites, elicit very low efficacy modulation but remains silent in most cases.

While the impact of the γ2 subunit remained limited in all observations so far (mean 1.1-fold with two-fold maximal change compared to the reference αxβ3 receptor), the delta subunit has been demonstrated to impact more profoundly on efficacy (i.e., 3.1-fold higher efficacy in α1β3δ compared to α1β3 for LAU 177; Supplementary Figure 5). As is the case for γ2, both the ligand identity and the α isoform determine the magnitude of the δ-sensitivity. It remains to be investigated experimentally how many additional binding sites, allosteric interactions between binding sites, and receptor properties drive these phenomena.

**FIGURE 4:**
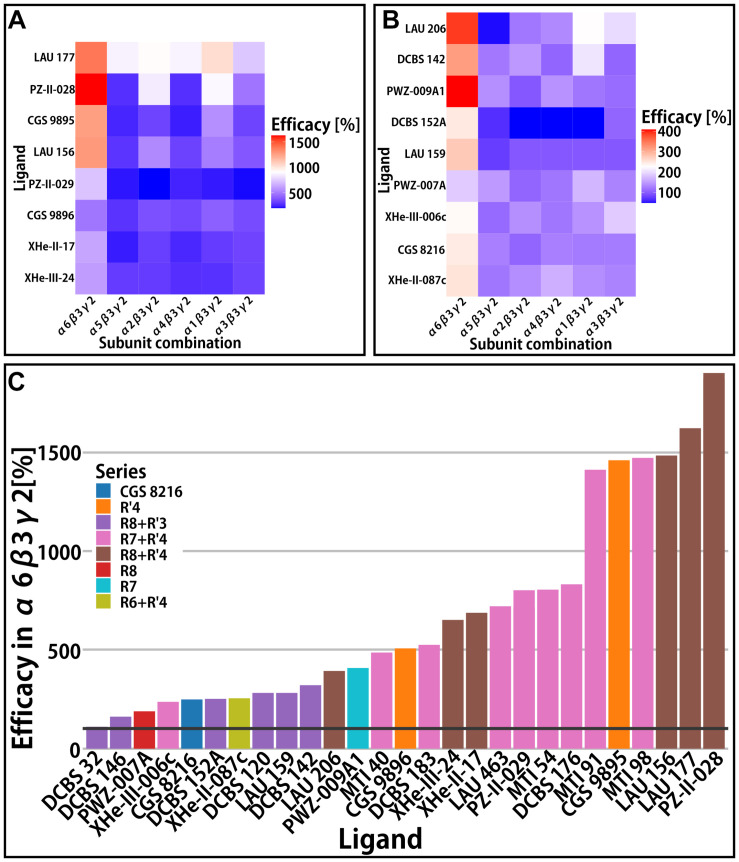
αxβ3γ2 efficacy. In panels **(A,B)** efficacy (% change of the reference GABA current at 100%) is presented in the form of heatmaps for the compounds with data available in all α-subunits. **(A,B)** Rows represent different compounds, while columns represent the receptors; both the rows and columns are ordered by similarity. **(A)** Heatmap of compounds with high efficacies (all above 500%) and **(B)** heatmap of compounds with lower efficacies. **(C)** Efficacy of compounds in the α6β3γ2 receptor. Bars are colored by the compound series. A black horizontal line is placed on 100% to delineate the point of no modulatory effect.

While the γ2 subunit is ubiquitously expressed in mammalian brains, α isoforms show higher degree of regio-specificity (Pirker et al., 2000; Hortnagl et al., 2013), and thus represent promising targets for subtype specific drugs. Since binary receptors with α4 and α6 subunits feature low GABA currents (Mortensen et al., 2011), the influence of these two isoforms on efficacy was studied only in αxβ3γ2 receptors (Varagic et al., 2013a, b; Treven et al., 2018). Here we present aggregated efficacy data for αxβ3γ2 receptors from several studies (Ramerstorfer et al., 2011; Varagic et al., 2013a, b; Mirheydari et al., 2014; Simeone et al., 2017, 2019; Treven et al., 2018). Among the compounds with low or very low efficacy in α1β3 or α1β3γ2 receptors, several turned out to display functional preference for other receptor subtypes. Specifically, many compounds have high efficacy in α6β3γ2 receptors and comparatively low or nearly no efficacy in the remaining αxβ3γ2 receptors (Figure 4). The cumulative data reveals that several different substitution patterns can lead to α6 selectivity. The R7 and R7+R′4 series contain both unselective and α6-selective compounds (Varagic et al., 2013a; Simeone et al., 2019). Additionally, the R8+R′3 series features the whole range of unselective compounds, an α6 selective compound (LAU 159), and with DCBS 152A a compound which exerts a mixed NAM/PAM profile (Figure 4 and Supplementary Figure 6). Only low to moderate efficacy compounds act silently in the off-target receptors. In contrast, compounds with very high efficacy tend to be α6-preferring, but display also moderate or high modulatory effect in all other isoforms (Figure 4 and Supplementary Figure 6).

In all subunit combinations tested in the experimental studies, a broad range of efficacies was observed. An aggregated view of the data in the light of compound series indicates that no particular substitution pattern shows strong tendencies for α-selectivity, or for (near) silent interactions with any subunit combination. As was discussed previously, substituents on rings A and D impact non-linearly on efficacy (Varagic et al., 2013b), and this is confirmed by the re-analysis of additional datasets. Thus, to identify a path forward for optimization of subtype profiles, complementary insight is needed.

### Binding Site Usage

**FIGURE 5:**
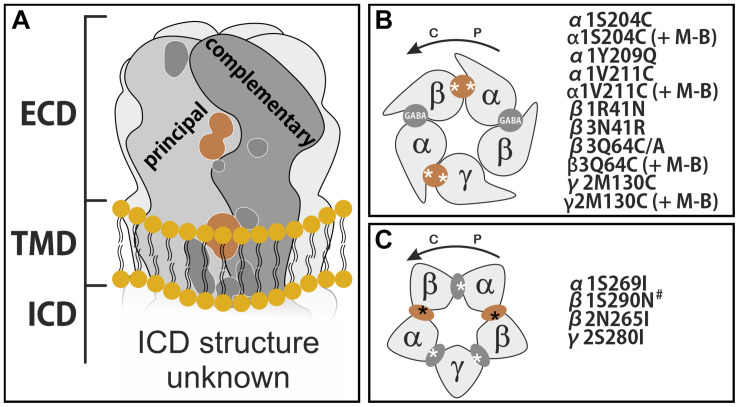
Binding sites on GABA_A_ receptors and sites proposed to be used by PQs. **(A)** Schematic side view of the membrane spanning receptor, with all so far described binding sites shown on a representative interface. Sites discussed in this work are depicted in light brown. ECD stands for extracellular domain, TMD for transmembrane domain and ICD for intracellular domain. **(B)** Schematic top view of the receptor ECD consisting of α, β, and a γ subunit. GABA sites are labeled. The ECD α+/γ2– site and the ECD α+/β– site are established extracellular PQ sites. **(C)** Schematic view of a plane through the upper TMD at the level of the binding sites used by e.g., etomidate. The unique pockets are at TMD β+/α– (two etomidate sites), TMD α+/β–, TMD γ+/β– (both barbiturate sites) and TMD α+/γ2–. In panels **(B,C)**, the approximate localizations of the mutations are indicated by asterisks, and the mutants are listed. The curved arrows indicate the direction from principal to complementary, counterclockwise if viewed from extracellular. M-B refers to the cysteine reactive reagent MTSEA-biotin. The # marked mutant β1N290N corresponds to β1N265N, as both conventions (numbering with or without signal peptide) have been used in the literature.

As described in the introduction, PQs have been demonstrated to interact with a multiplicity of binding sites, as shown in Figure 5. Here we briefly review the details of the experimental evidence.

Radioligand displacement is a standard method to demonstrate usage of a known binding site, for which radioligands are available. Such data has been accumulated for ECD α+/γ2− for the case of PQs over many years (Zhang et al., 1995; Savini et al., 1998, 2001; Carotti et al., 2003; Knutson et al., 2018). In Table 3 we provide the Ki data available for the compounds we study here. Most of them display affinities in the nanomolar range, with the notable exceptions of LAU 462 (Simeone et al., 2017) and XHe-II-087c (Varagic et al., 2013a, b).

**TABLE 3:**
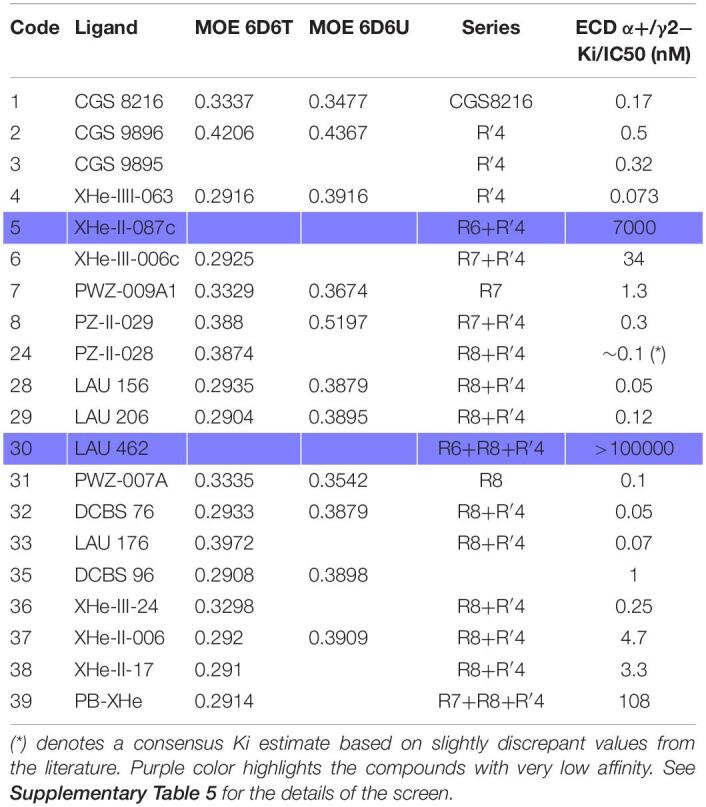
PQ pharmacophore screen 3.1 results from MOE (RMSD values) into the flumazenil-bound structures (PDB ID: 6D6T and 6D6U) with series information and the Ki/IC50 values (nM) as reported in the original papers.

(*) denotes a consensus Ki estimate based on slightly discrepant values from the literature. Purple color highlights the compounds with very low affinity. See Supplementary Table 5 for the details of the screen.

For all other sites, which can be targeted by PQs, radioligands are not yet available. Thus, mutational analysis is commonly used to test binding site hypotheses. We list all the described GABA_A_ receptor mutations for PQs in Supplementary Table 7. The ECD α+/β− site was first described in 2011 (Ramerstorfer et al., 2011), when a mutation-based so-called steric hindrance approach was employed to demonstrate a pronounced loss of modulation by CGS 9895 upon covalent modification of cysteines placed at selected positions in the ECD α1+(α1V211C) and ECD β3− (β3Q64C) half pockets (Supplementary Table 7 and Supplementary Figure 7). In later studies similar findings for ECD α1+/β3− were presented for PZ-II-028 and XHe-II-087c (Varagic et al., 2013b), and LAU 177 (Mirheydari et al., 2014). Furthermore, for PZ-II-028, PZ-II-029, and CGS 8216 steric hindrance at ECD α6+/β3Q64C-MB was demonstrated (Varagic et al., 2013a). Mutational studies provided further evidence of α1Y209 involvement in the modulatory effects of CGS 8216, CGS 9895, CGS 9896, LAU 176, and LAU 177 (Maldifassi et al., 2016; Supplementary Table 7 and Supplementary Figure 7). Interestingly, α1Y209Q exerted a variable impact on these compounds (Supplementary Table 1; Maldifassi et al., 2016). Contributions of the complementary part of the ECD α+/β− interfaces were not only probed by steric hindrance, but also investigated through “conversion mutants.” β3N41R – a substitution of an amino acid for a corresponding β1 isoform amino acid – was utilized to probe the influence of this residue on the efficacy of compounds, which display variable degrees of functional β preferences (Simeone et al., 2017; Figure 2B, Supplementary Table 7 and Supplementary Figure 7). The results, obtained with the mutant receptor demonstrated the impact of this position on the subtype-specific potency and efficacy of six ligands from the R8+R′4 series (Supplementary Table 1). In another line of work γ2 residues were introduced to the β3 complementary face, showing Q64A to have a big impact on the potency of CGS 9895 (Siebert et al., 2018a).

Transmembrane domain sites were investigated so far only in two studies. CGS 9895 displays low efficacy in α1β1 compared to α1β3, and was seen to lose efficacy in the conversion mutant β3N265S [N290S according to the nomenclature with the signal peptide included, (Ramerstorfer et al., 2011)]. A more extensive investigation for CGS 9895 and LAU 177 probed the influence of the homologous position at TMD β2+(N265I), α1+(S269I), and γ2+(S280I) expressed either individually with two wild type subunits, or all three mutated subunits in combination (Maldifassi et al., 2016). Thus, for CGS 9895 and LAU 177 mutational analysis at multiple TMD binding sites indicates that the ECD α+/β− interface is not the only contributor to the efficacy of modulation. Instead, net modulation was also influenced to some degree by all the sites at the upper TMD interfaces. These sites are otherwise known for conveying the action of ligands such as etomidate, barbiturates, or avermectin (Lynagh and Lynch, 2012; Maldifassi et al., 2016; Iorio et al., 2020). In conclusion, the combined experimental evidence demonstrates that PQs depending on the details of the substitution patterns are ligands of at least five distinctive binding sites on a given GABA_A_ R pentamer: The ECD α+/γ2− (benzodiazepine), the ECD α+/β−, the TMD β+/α− (etomidate), and the TMD α+ and γ+ containing interfaces (Figure 5). This has been underappreciated, and since it is now clear that total modulation could come from both the ECD α+/β− site together with multiple TMD sites, it is not surprising that we see almost no correlation between ligand structure variations and variations in efficacy profiles.

The ECD principal component of the α subunits is unique for each isoform, making ECD sites preferred for selective targeting over the more conserved TMD sites (Supplementary Figure 2). Though PQs exert at least some of their effect through ECD α+/β− sites, the data suggests the total efficacy for many of them could come from multiple sites. All in all, this points to the clear need to develop ligands specific for the ECD α+/β− sites (Sieghart et al., 2012). Supplementary Figure 2 depicts an alignment of the ECD segments contributing to the sites that can be targeted selectively. Furthermore, it shows the TM1 (transmembrane helix 1) contribution of α subunits to the etomidate site, which lacks variable amino acids. Supplementary Figure 7 displays in a representative 3D structure rendering the positions in the ECD α1+/β3− site where mutational analysis was published. In order to steer ligand properties toward the desired profiles, structure activity relationship (SAR) models can be employed as depicted in Supplementary Figure 8. Individual SAR models would be built for the desired site, as well as for each unwanted site.

### Computational Predictions of Binding Modes at the ECD and TMD Sites

If bound state structures are available, SAR model building and thus ligand design can be structure based and ligands with distinctive ligand-protein interactions can be modeled separately. Even in the absence of experimental structures with PQs in any of the before mentioned sites, diverse computational methods can be employed. For ligand-bound pockets, pharmacophore matching offers rapid throughput, while computational docking is needed for binding sites in apo states. Thus, pharmacophore matching was employed for two of the PQ site candidates. Several structures have been released with benzodiazepines at the high affinity ECD α+/γ2− site (Zhu et al., 2018; Masiulis et al., 2019), and in one of them diazepam also occupies the TMD β3+/α1− site (Masiulis et al., 2019). By using these, structure-based pharmacophore methods can be applied to the high affinity ECD α1+/γ2−, and to the TMD β3+/α1− site. After the study was completed, an etomidate-bound structure also became available (Kim et al., 2020).

Pharmacophore methods aim to identify ligand features that drive interactions with a specified target (see Supplementary Figure 1; Wolber and Langer, 2005; Sanders et al., 2012). Overlay-based methods such as LigandScout and RMSD-based methods as implemented in MOE tend to be quite complementary in performance (Sanders et al., 2012). Thus, both were used as described in the “Materials and Methods”. In the screens with default parameters (screens 1.1 and 1.2, Supplementary Table 2) matches of several PQs with the high affinity flumazenil-derived pharmacophores were observed. Screens into the diazepam and alprazolam bound states, in contrast, yielded almost no matches. Of note, flumazenil does not share a common binding mode with diazepam and alprazolam (Elgarf et al., 2018; Zhu et al., 2018; Masiulis et al., 2019). Thus, the flumazenil bound structure represents a pharmacophore distinct from the diazepam and alprazolam bound states. Like flumazenil, PQs are mostly silent binders (Ramerstorfer et al., 2011), making the results consistent with the functional similarity, and weakly suggestive of different pocket regions mediating silent versus PAM ligands.

Screens 1.1 and 1.2 into the TMD-bound diazepam pharmacophore resulted in nearly no matches for PQs. For five known binders of the TMD β3+/α1− site, four matches were seen in screen 1.1, while screen 1.2 only matched midazolam, which is expected based on the high similarity with diazepam (Supplementary Table 2).

In the further analysis we focused on the use of MOE, which has been suggested to perform well for binding mode prediction, while LigandScout is the better choice for enrichments (Sanders et al., 2012). To design unwanted binding out of a scaffold, binding mode hypotheses are needed. In a next step we thus tested a variety of screening parameters in MOE for the ability to recapitulate structurally diverse binders of the TMD β3+/α1− site, and to identify settings which recapitulate more of the high affinity binders at the flumazenil-bound ECD α1+/γ2− site. For this, etomidate, loreclezole, mefenamic acid, midazolam, and valerenic acid were utilized as known binders, and the Z-drugs zolpidem, zaleplon and zopiclone were used as non-binders for the TMD. In a series of eight screens (2.1 to 2.8, see Supplementary Tables 3, 4) the influence of exclusion sphere and ligand shape radii was explored.

In the benchmarking screens we monitored the hits among high affinity binders to the flumazenil pharmacophore for the ECD α1+/γ2− site. Settings with exclusion sphere usage enabled (radius at the default value) as used in screen 3.1 performed well for high affinity PQs at the ECD α1+/γ2− site (Table 3 and Supplementary Table 5). The 6D6T-derived pharmacophore was matching 17 of 18 high affinity PQs and rejecting the compounds with Ki > 7000 nM.

For the diazepam-bound TMD β3+/α1− site in 6HUP, different parameters needed to be optimized to correctly match etomidate, loreclezole, mefenamic acid, midazolam and valerenic acid to the site, while retaining the rejection of Z-drugs. In brief, optimizing ligand shape radius, and disabling exclusion spheres proved beneficial as documented in detail in Supplementary Tables 3, 4. After this study was completed, an etomidate-bound structure of the TMD β3+/α1− site also became available (Kim et al., 2020). We thus added screen 4 (Supplementary Table 6), in order to have a side-by-side comparison of the diazepam-derived and the etomidate-derived pharmacophore models of this site. In both models the positive controls were correctly matched while the Z-drugs were rejected, in line with experimental findings. At settings which match the known binders and reject Z-drugs, up to 17 PQs also match (Supplementary Tables 5, 6). Due to the lack of experimental data, which is available only for two PQs (Maldifassi et al., 2016) the individual predictions from the screens at the TMD site cannot be verified beyond the observation that pharmacophore screening predicts binding of PQs to this site based on both the diazepam-bound and the etomidate-bound 3D structures.

**FIGURE 6:**
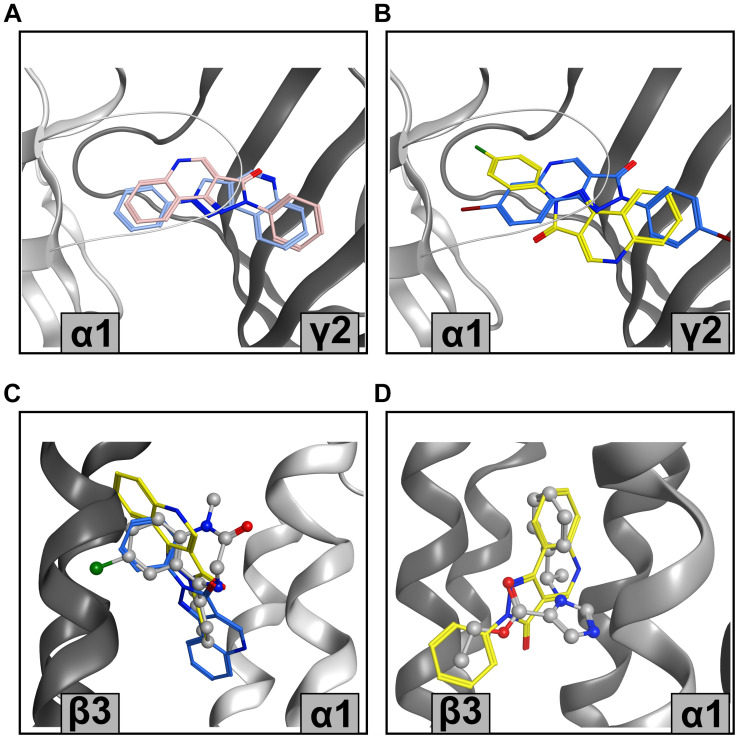
Representative PQ bound states from the MOE pharmacophore screen **(A)** Structure-based pharmacophore predictions into the ECD α1+/γ2– flumazenil-bound structure (PDB ID: 6D6T) from LigandScout (blue) and MOE (pink) for CGS 8216. **(B)** Screen 3.2 predictions for XHe-III-006c (blue) and CGS 9896 (yellow) in ECD α1+/γ2– (PDB ID: 6D6T). **(C)** Screen 3.2 results for CGS 8216 (blue) and LAU 165 (yellow) in TMD β3+/α1– diazepam-bound structure (PDB ID: 6HUP); diazepam is depicted in balls and sticks. **(D)** CGS 8216 (yellow) results from screen 4.1 in etomidate-bound TMD β3+/α1– with ligand shape radius set on 2.5 Å. Etomidate is shown in balls and sticks representation (PDB ID: 6x3V).

The highly satisfactory performance of screen 3.1 for the high affinity interactions with the ECD α1+/γ2− site justifies the use of the predicted binding mode models toward structure-based drug design. Interestingly, PQs do not show a single binding mode resulting from the best performing screen, but a diversity of binding modes (Figures 6A,B). Of the predicted binding modes, one overlaps well with what has been proposed previously based on docking and MD simulations (Siebert et al., 2018b) (see pink pose in Figure 6A).

The TMD site matches cannot be verified due to the lack of experimental data, but some insights can still be gained. Again, we observe a diversity of binding modes resulting from the pharmacophore matching (Figures 6C,D). This is not surprising in the light of enabling feature omission, and a ligand shape radius allowance which permits the matching of the chemically highly diverse known binders. Of note, the recently released diazepam-bound barbiturate site at the TMD γ2+/β2− site features a binding mode of diazepam very different from the one in the etomidate site (Kim et al., 2020).

The binding mode hypotheses derived from the screens into the high affinity site are consistent with all available experimental evidence, and thus a valid starting point to explore avenues for the reduction of affinity to this site. The large hydrophobic tBu group in R6 works well, but renders the compounds poorly soluble. Thus, a structure-guided approach to novel derivatives should accelerate ligand development. In the case of the interactions with the TMD site, more experimental observations are needed to rank the predicted structures.

### Docking

**FIGURE 7:**
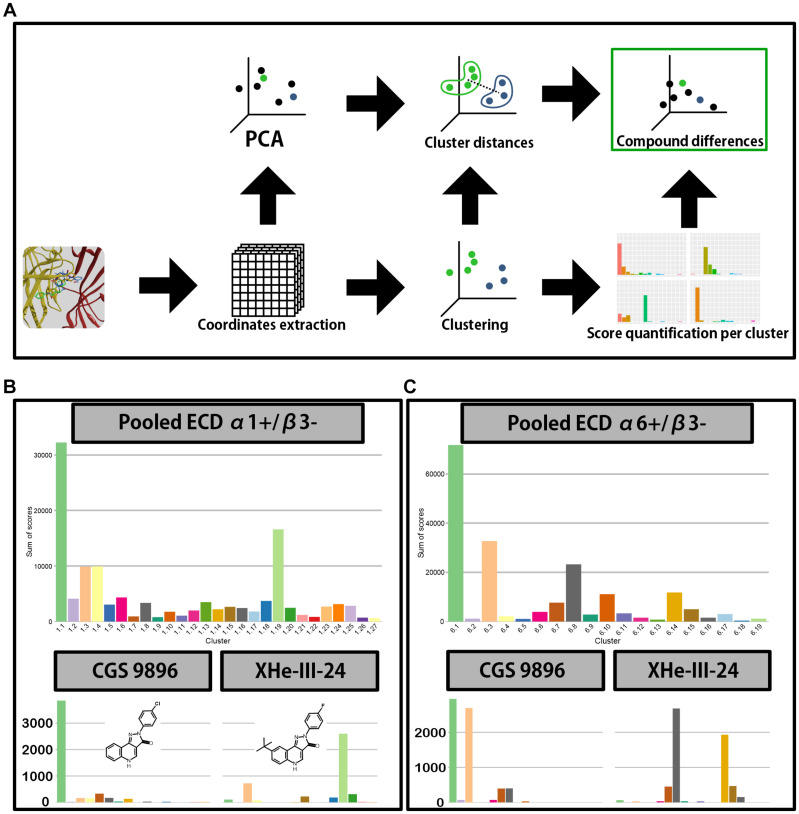
Docking analysis pipeline and clustering results. **(A)** A scheme depicting the analysis workflow of docking results. First the polar coordinates get extracted from the generated docking poses. The coordinates are then used for principal component analysis and clustering; the results from both are in turn used to calculate distances between centroids of clusters and to produce a distribution of score across the clusters for all compounds. The score distributions and between-cluster distances are then used by Earth mover’s distance calculation to compare the compounds. **(B,C)** Distribution of score across the clusters for ECD α1+/β3– **(B)** and ECD α6+/β3– **(C)** docking. Top graph depicts the score weighted distribution when all the compounds are pooled together, while the bottom graphs depict the distribution for two representative compounds–CGS 9896 (left) and XHe-III-24 (right). Compound structures are depicted in panel **(B)** inserts.

To generate structural hypotheses for PQ interactions with the target site of interest, we performed docking of 30 compounds (Table 1) into ECD α1+/β3− and ECD α6+/β3− pockets. The docking runs were analyzed as shown in Figure 7A and described in the “Materials and Methods” section. In short, extracted coordinates were used for dimensionality reduction and clustering. All pose clusters that were populated by less than 10 poses of any individual compound were discarded. This resulted in 27 pose clusters in case of ECD α1+/β3− (1.1–1.27, Figure 7B upper) and 19 in case of ECD α6+/β3− (6.1–6.19, Figure 7C upper). As pose scoring represents a quantitative measure for “pose fitness,” we switched to score-weighted population analysis in the remainder of the analysis. This meant summing up the scores of poses, residing in each individual cluster – be it for pooled data, or when looking at individual compounds.

In both binding sites the cumulative pooled score accumulates in a single pose cluster, with the following cluster having approximately half the accumulated score (Figure 7B) or less (Figure 7C). Thus, the pooled data could indicate an overall preference for a single binding mode for the compound class (common binding mode hypothesis). On the other hand, the individual compounds might not follow the trends observed in the pooled data (multiple binding modes hypothesis). Indeed, when looking at the individual compounds we see a high level of variation in the distribution of score between the pose clusters (Supplementary Figures 10, 12). For example, in both binding sites CGS 9896 shows preference for pose clusters 1.1 and 6.1–clusters with the highest aggregated score. On the other hand, in case of XHe-III-24 there is very little accumulation of score in pose clusters 1.1 and 6.1. It rather prefers pose cluster 1.19 in case of α1+/β3−, or 6.8 in α6+/β3− (Figures 7B,C).

**FIGURE 8:**
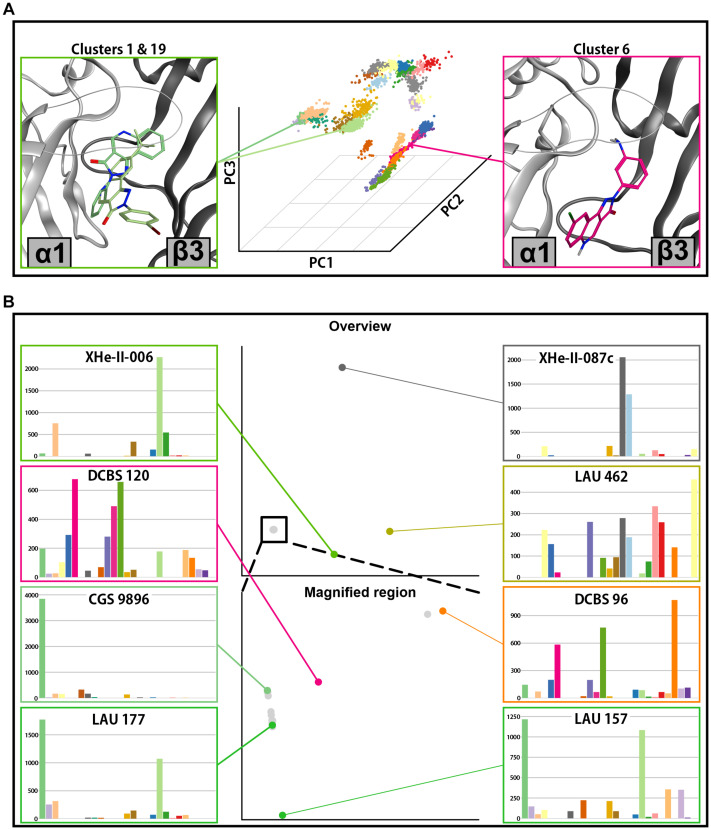
Docking analysis results for ECD α1+/β3–. **(A)** In the center posing space for ECD α1+/β3– docking is depicted. Each point on the graph represents a single pose from all docking runs in this binding site. The position of the points is defined by the first three principal components of the PCA analysis. The clusters are shown in different colors. Representative poses for two clusters with highest accumulated score are depicted on the left side, while a representative pose from the pose cluster 1.6–highest accumulated score in the other subset of pose clusters, is depicted on the right. Clusters 1 (darker green) and 19 (lighter green) are depicted on the left, while cluster 6 (magenta) is depicted on the right. **(B)** Full compound embedding for ECD α1+/β3– is shown in the center top. The space in the square is shown enlarged in the center-bottom. Each point on the two graphs represents a single compound. The score distributions of the selected compounds are shown on both sides of the two central graphs.

**FIGURE 9:**
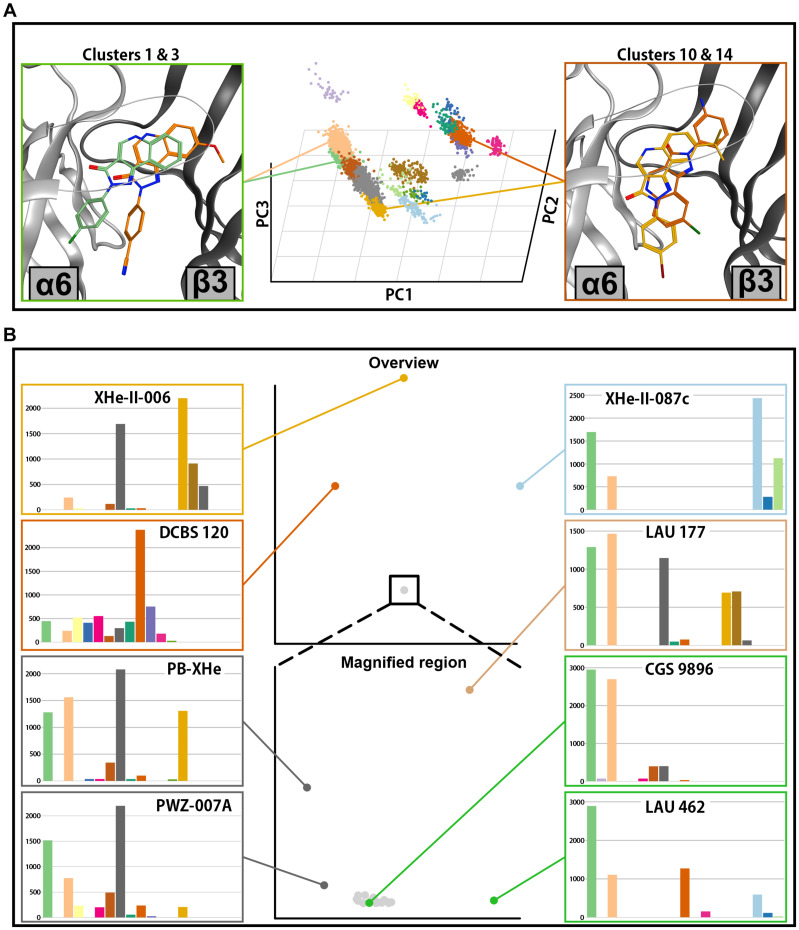
Docking analysis results for ECD α6+/β3–. **(A)** In the center, the posing space is depicted. Each point on the graph represents a single pose from all docking runs in this binding site. The position of the points is defined by the first three principal components of the PCA analysis. The pose clusters are shown in different colors. Representative poses for two pose clusters with the highest accumulation of score are shown on the left side (cluster 1 in green and cluster 3 in orange), while the representative poses from two clusters with high accumulated score only in a small subset of compounds are depicted on the right (cluster 10 in dark orange and cluster 14 in yellow). **(B)** Full compound embedding for ECD α6+/β3– is shown in the center top. The space in the square is shown enlarged in the center-bottom. Each point on the two graphs represents a single compound. The score distributions of the selected compounds are shown on both sides of the two central graphs.

Sum of score distributions for all compounds are provided in Supplementary Figures 10, 12. Both ECD α1+/β3− and ECD α6+/β3− pose spaces show substantial diversity (Figures 8A, 9A). In both cases the full space can be divided in two subsets, defined by the general orientation of the molecule. Interestingly, the mean pose score in the subsets also differs significantly for both α1+/β3− (*p*-value 1.428 × 10^–06^) and α6+/β3− (*p*-value 6.94 × 10^–11^). When looking at the sum of score across the pose clusters with all the compounds pooled together (Figures 7B,C) the highest scoring cluster from the less preferring subset (1.6 for ECD α1+/β3− and 6.10 for ECD α6+/β3−) does not seem to be a contender for a single best candidate binding mode. On the other hand, both cluster 1.6 and 6.10 seem to be preferred in compounds with an amino group on ring D (Figures 8B, 9B–DCBS120, DCBS 96; Supplementary Figures 10, 12). When looking at the position of the molecule in the pocket for these two clusters and the other clusters in the same subsets, we observe a 180° turn compared to the clusters from the other subsets (Figures 8A, 9A). Clusters 1.1 and 6.1 have most accumulated score (Figures 7B,C). Interestingly, they are both defined by a similar orientation of the molecule in the pocket (Figures 8A, 9A and Supplementary Figure 14). The per-compound score distributions thus support the multiple binding mode hypothesis.

The posing space together with the sum of score distributions across the pose clusters imply that individual ligands show distinctive profiles: unambiguous posing into highly populated clusters, unambiguous posing into rare clusters, and ambivalent posing. In a next step this was analyzed in more detail. The distribution of scores across the pose clusters and the distance between the clusters on the dimensionality reduced space can be used to compare the compounds between each other. The used algorithm (Earth mover’s distance) produces pairwise distances between the compounds, which in turn can be used to produce an embedding – a visual representation of dissimilarity. Figure 8B depicts such an embedding for the ECD α1+/β3− docking run, while a detailed view is given in Supplementary Figure 11. In the full embedding space are four distinct clusters, where the majority of compounds occupy one large compound cluster. This most populated compound cluster can be seen to fragment further in a magnified view (Figure 8B and Supplementary Figure 11). Most compounds follow a central line, with a branch that contains compounds with an amino group on ring D (DCBS 120, LAU 206, and DCBS 96). The compounds on this branch are ambiguously posed, with a relative decrease of score in pose cluster 1.6 and an increase in pose cluster 1.24. The central branch at one end features the non-ambiguous case of the pose cluster 1.1-preferring CGS 9896, and aligns on an axis with compounds that show mixed preferences and a decreasing pose cluster 1.1 to 1.19 ratio, with LAU 157 on the most distal position and nearly equal scores accumulating in these two pose clusters (Figure 8B and Supplementary Figure 11).

The distant compound clusters contain a total of six compounds. XHe-III-0087c and LAU 462 populate their own unique compound clusters. XHe-II-24, XHe-II-17, and XHe-II-006, the only three compounds with tBu on the R8 position, populate a compound cluster with a strong preference for pose cluster 1.19 (Figure 8B and Supplementary Figures 10, 11). Generally, the compounds which are furthest from the most populated compound cluster all contain a tBu rest on ring A, and account for 5 out of 6 largest compounds in the considered set.

**FIGURE 10:**
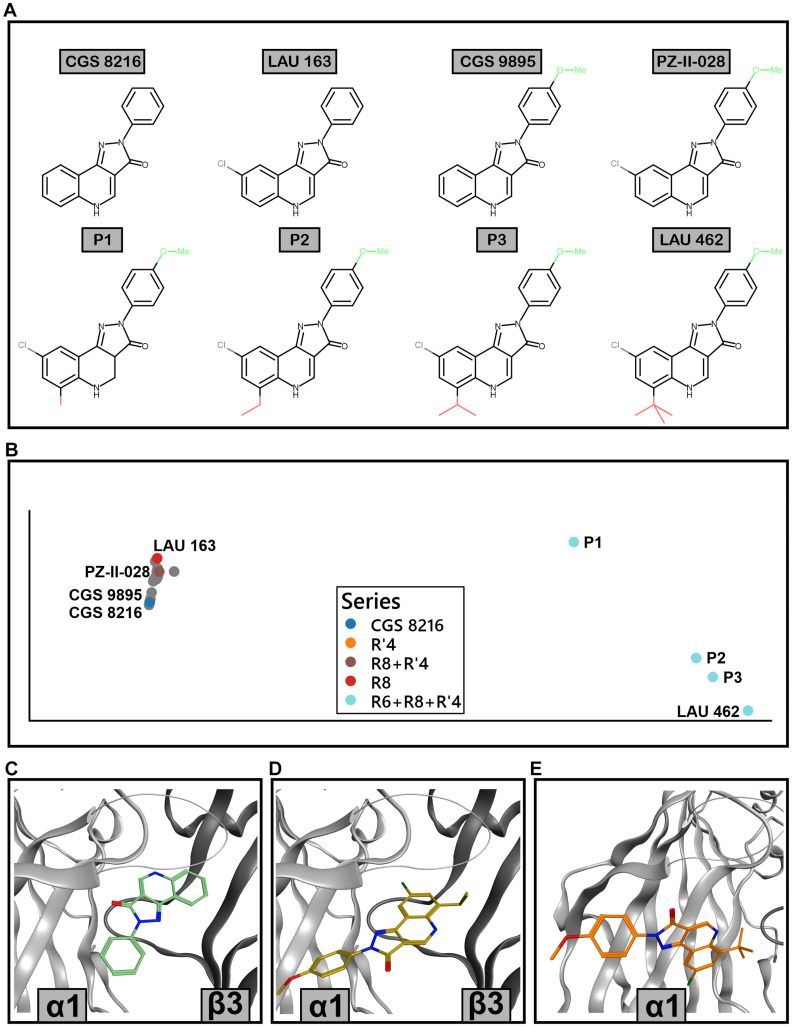
Extended ECD α1+/β3– docking analysis with R6-substituted compounds. **(A)** 2D structures of the compounds that were analyzed in detail; their poses are marked with color in panel **(B)**. P1, P2, and P3 are additional compounds, with increasing size of the residue in position R6. **(B)** Compound embedding of the extended docking results. Colored dots show the compounds from panel A while gray dots represent other compounds. **(C)** Best scored pose of CGS 8216 from pose cluster 1.1. **(D)** Best scored pose of P3 from pose cluster 1.4. **(E)** Best scored pose of LAU 462 from pose cluster 1.27.

To explore how modifications in position R6 influence the posing space, we added three compounds which fall into a series between PZ-II-028 and LAU 462 (Figure 10A). This results in a compound embedding in which LAU 462 is no longer isolated far from other compounds, but the endpoint of a separate branch, on which the compounds are positioned according to the increasing size of R6 substituent (Figure 10B). Of note, bulk in R6 does not seem to induce a single new binding mode, but leads to multiple candidates (Figures 10C–E). The combined results indicate that bulk in both R6 and R8 induce critical branching points.

The compound space for ECD α6+/β3− docking looks similar to the ECD α1+/β3− (compare Figures 8B, 9B). A large compound cluster fragments further when zooming into it, and three compound clusters are more distal to this aggregate. The main compound cluster shows a major preference for pose clusters 6.1 and 6.3 (Figure 9B, lower part). Of those, pose cluster 6.1 is very similar to 1.1 (Supplementary Figure 14). In the score distributions of the α6+/β3− results we observe a higher degree of ambiguity between the most populated pose cluster 6.1 and the relatively similar 6.3 (Supplementary Figure 12).

There are some notable differences between compound space in α6+/β3− versus α1+/β3−. The position of LAU 462, which in α1+/β3− populated a unique compound cluster with very ambiguous posing, is in α6+/β3− near the most populated compound cluster with a posing preference for cluster 6.1. As in α1+/β3−, the group of compounds with tBu on R8 (XHe-II-006, XHe-II-17, and XHe-III-24) are again distal to the most populated compound cluster, albeit less far away in compound space. In contrast, the three compounds with amino group on ring D (DCBS 96, DCBS 120, and LAU 206) are positioned further away from the main cluster compared to ECD α1+/β3−. Comparing the compound spaces with branching graphically shown (Supplementary Figures 11, 13) provides an overview of the similarities and differences.

**FIGURE 11:**
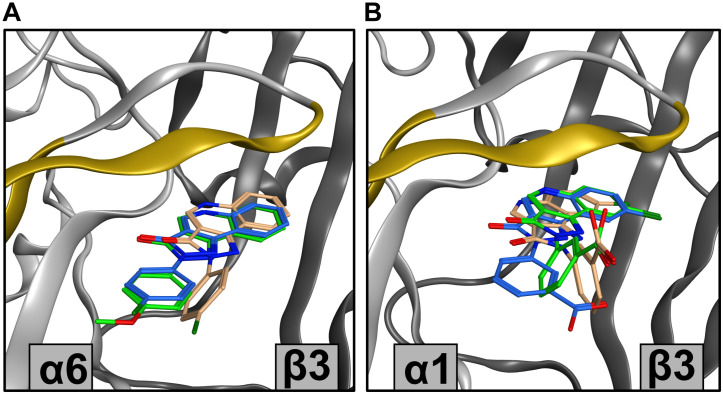
Showcase of representative poses from docking results. **(A)** Top ranking poses from clusters with highest sum of scores for CGS 9895 and CGS 9896 from ECD α6+/β3– docking. For CGS 9895 cluster 1 is depicted (green), while for CGS 9896 clusters 1 (blue) and 3 (orange) are depicted. The variable amino acids in α subunits on loop C, and strands 9 and 10 are marked in yellow. **(B)** Top ranking poses from several clusters for DCBS 152A docking. From ECD α1+/β3– docking clusters 1 (green) and 3 (orange) are depicted, while from the ECD α6+/β3– docking only cluster 1 (blue) is viewed. The variable amino acids in α subunits on loop C, and strands 9 and 10 are marked in yellow.

Analysis of posing space and compound embedding indicates a complex structure-activity landscape of the PQ scaffold with multiple branching points. The two pockets display some common pose clusters and some differences, which manifest in distinctive compound embeddings. In an effort to relate the structural predictions to experimental observations, we examined several compounds in more detail, searching for potential structural hypotheses for their efficacy profiles in different receptor subtypes. CGS 9895 and CGS 9896 display similar efficacy in α1-containing receptors. However, CGS 9896 is nearly unselective while CGS 9895 exerts high efficacy in the α6β3γ2 receptor (Figure 4B, Supplementary Figure 6, and Supplementary Table 1). In the posing space, the score distributions indicate that both compounds unambiguously favor pose cluster 1.1 in the ECD α1+/β3− docking (Supplementary Figure 10). In contrast, CGS 9895 shows strong, but not exclusive preference for the similar pose cluster 6.1 in the ECD α6+/β3− docking, while CGS 9896 populates pose clusters 6.1 and 6.3 in an ambivalent fashion (Supplementary Figure 12). While these correspond to fairly similar binding modes for the fused ring system, ring D is posed quite distinctively and thus can be expected to make different interactions with the protein (Figure 11A and Supplementary Figure 15). This structural hypothesis for the observed difference in efficacy preference would require experimental testing to confirm or reject it, but further support comes from another compound with a similar score distribution. PWZ-009A1 also populates pose cluster 6.3, and also has relatively modest efficacy in the α6β3γ2 receptor (Figure 4B and Supplementary Figure 6). Thus, two ligands with different substituent patterns share posing space preferences and efficacy profiles, a finding which should be explored further in a systematic study of derivatives.

Probably the most striking efficacy profile has been reported for DCBS 152A, which exerts negative allosteric modulation in αxβ3γ2 (*x* = 1, 2, 4, 5), is silent in α3β3γ2, and a positive allosteric modulator in α6β3γ2 (Treven et al., 2018; Supplementary Figure 6). DCBS 152A is an ambivalently posing compound in ECD α1+/β3−, with a slight pose cluster 1.1 preference (Supplementary Figure 10) and also ambivalently posing in ECD α6+/β3−, with a preference for pose cluster 6.1 (Supplementary Figure 12). The substituent on ring D is in the R′3 position, which could pose in two rotationally equivalent positions, but interestingly only one of these possibilities occurs in the posing space. Furthermore, pose cluster 6.1 from the ECD α6+/β3− has the ring D consistently turned 90° compared to the pose clusters 1.1 and 1.3. As is the case for CGS 9895 and CGS 9896, the main difference in the alternative pose clusters mainly impacts on ring D position (Figure 11B and Supplementary Figure 16). In turn, the substituent contacts loop C in distinctive ways. This structural hypothesis is thus very consistent with the fact that each alpha isoform features a unique sequence signature in loop C, as depicted for the case of α1 and α6 in the Supplementary Figure 2 (Puthenkalam et al., 2016). In this showcase, the intriguing observations for a single compound suggest a follow up.

All in all, the exhaustive sampling and analysis of posing space, score distributions across pose clusters and the identification of branching points in compound embedding show the potential to generate interesting structural hypotheses. Putative correlations with experimental findings are still somewhat limited for compounds with ambiguous score distributions and a more diverse posing space, but will be highly informative for the design of decisive experiments. The analysis of the docking data can readily be applied to much larger compound sets in future studies.

## Discussion

Historically, PQs have been developed as ligands of the high affinity benzodiazepine binding sites (Iorio et al., 2020; Vega Alanis et al., 2020). In turn, a number of derivatives such as CGS 8216, CGS 9895, and others were used in pre-clinical research. Based on their *in vivo* profiles they were considered as a compound class exerting positive, silent (antagonistic) or negative allosteric modulation by their interaction with the benzodiazepine binding sites, with the substitution pattern defining the efficacy profile. Additionally, a tritiated version of CGS 8216 has been widely utilized as a radioligand (Boast et al., 1985).

Due to promising *in vivo* efficacies as anticonvulsant and anxiolytic drugs, the toxicology of the compound class was assessed and found to be very favorable. Although these initial developments did not result in any clinical drug, the interest has recently been revived due to the discovery of a “mPQ binding site,” which is located at extracellular α+/β− interfaces (Sieghart et al., 2012). In turn, the description of PQs with α6-selective effects stimulated pre-clinical studies aimed at novel therapeutic principles targeting α6-containing GABA_A_ receptors in the cerebellum (Chiou et al., 2018) and in the trigeminal nociceptive pathway (Fan et al., 2018; Vasovic et al., 2019). As is the case for previously tested compounds of this class, these novel α6-selective PQs also display excellent toxicological properties in pre-clinical assays (Knutson et al., 2018).

Further improvement of this promising class of GABA_A_ receptor modulators has been achieved when trying to overcome challenges in their physicochemical and metabolic properties (Knutson et al., 2018). In turn, this resulted in an ever-growing availability of functional and mutational data for novel derivatives of this scaffold.

Re-analysis of all aggregated data provides some insights concerning ligand features that may drive desired functional selectivity. The initially described R8+R′4 substituted compounds show no tendency toward α selectivity, while exhibiting limited β selectivity. Adding bulk on position R6 is a promising path to eliminate ECD α+/γ2− affinity, and it seems to induce different binding mode preferences leading to a branch in the PQ-SAR landscape (see Figure 10). The less explored R7+R′4 substituted compounds led to the initial description of α6-preferring compounds, but too few compounds exist to derive robust SAR models. The desired loss of efficacy in α1-containing combinations was incomplete for most cases in this series. Generally, compounds with very high efficacy tend to be less selective. In contrast, compounds bearing substituents in R8+R′3 tend to display lower efficacy but combined improved selectivity, as exemplified by LAU 159 (Figure 4; Treven et al., 2018). Moreover, this series delivered the first compound with a mixed NAM/PAM profile. This compound interacts with αxβ3γ2 receptors with a completely novel profile of isoform specificity: as NAM in the isoforms α1, 2, 4, 5, while being silent in α3 and PAM in α6.

An emerging challenge in the exploitation of promising subtype profiles is the promiscuity with which these compounds bind to several distinctive sites on the receptors. This is not unique to the PQ chemotype, and has also been observed consistently for benzodiazepines (Walters et al., 2000; Maldifassi et al., 2016; Masiulis et al., 2019; Iorio et al., 2020; Kim et al., 2020). To take advantage of the variable ECD α+/β− sites we aim to advance the understanding of PQ SAR in two steps that ideally can be accomplished sequentially: (1) bound state hypotheses of promising subtype selective sub-series provide the features which are needed to drive recognition and selectivity at the desired site; (2) the off-target interactions are designed away by the introduction of features that do not interfere with ECD α+/β− recognition. Supplementary Figure 8 depicts in more detail on which front more experimental data is needed to guide computational efforts, and how these can in turn be employed to design informative ligands for testing binding mode hypotheses.

Toward the first aim we performed an analysis of the PQ posing space for 30 PQ derivatives. We have exhaustively explored the posing space for 30 ligands in two ECD pockets and applied for the first time a modified workflow on docking results (Figure 7A) to characterize score-weighted posing space. The workflow which we present here is aimed at sufficiently large runs to avoid artifacts from undersampling of posing space (Supplementary Figure 9). We found strong evidence for multiple branching points, with the bulk of the investigated ligands showing clear preference for a well-defined binding mode (Figures 8, 9 and Supplementary Figures 11, 13), and groups of ligands with clear preference for vastly different binding modes. As would be expected, sterically demanding substituents can drive changes in posing preferences, but more subtle factors also lead to ambiguous posing or branching. Thus, the computational results provide evidence for a “multiple binding mode hypothesis.” A recent study based on the common binding mode hypothesis (Singh and Villoutreix, 2020) presents an interesting contrast to the findings presented here. A comparison between our findings and (Singh and Villoutreix, 2020) is in Supplementary Figure 17 and its legend.

By combining computational docking with mutational analysis and systematic ligand variations our lab predicted that molecules of the benzodiazepine type should bind at their high affinity ECD α+/γ2− site with different binding modes (Elgarf et al., 2018; Siebert et al., 2018a). This was confirmed by the recent structural data (Masiulis et al., 2019; Kim et al., 2020). Similar to the benzodiazepines, our computational analysis of PQ-binding to the high affinity ECD α+/γ2− predicts multiple binding modes. When focusing on removing the interaction of future compounds with this unwanted binding site further systematic experimental data is needed to gain insight in how SAR landscape looks at both ECD binding sites. This would in turn be necessary to understand where the design leaves room for steering interactions at both sites into opposite directions.

Last but not least, the interactions with the TMD site need to be understood. Not only PQs, but also molecules of the benzodiazepine type show promiscuous binding at TMD sites (Masiulis et al., 2019; Kim et al., 2020). Our computational analysis suggests that interactions with the TMD sites are also not limited to distinct binding modes, which is highly plausible given the large hydrophobic surface and conformational flexibility of these sites (Puthenkalam et al., 2016). The accumulated data for LAU 177 suggests that PQs with very high efficacy might exert a substantial fraction of the total modulation via the TMD sites: much of the efficacy is lost by mutating β2N265 at the TMD β+/α− site (Maldifassi et al., 2016), but also upon steric hindrance at ECD α+/β− site (Mirheydari et al., 2014). While the extracellular site can mediate functional selectivity due to the variable binding site segments (Supplementary Figure 2), the TMD site is largely conserved across α isoforms. This may explain why the baseline of modulation is relatively high in all receptor subtypes for the compounds that exert strong effects via the TMD sites. With this finding as a starting point, it would be desirable to compare high efficacy and low efficacy PQs side by side concerning their ability to occupy TMD sites.

In spite of the increased abundance of data, it is still too scarce for the successful identification of essential features, which drive the interaction with the desired sites in the ECD, while avoiding unwanted interactions with the competing sites. Supplementary Figure 8 and Supplementary Table 7 indicate that the data most crucially needed from *in vitro* studies is the potential use of the TMD sites. The magnitude of the problem derived from binding to this site cannot be estimated with currently published data for only two compounds. Individual ligand prototypes from so far underexplored substitution series should be very valuable, as long as all the binding sites are monitored experimentally. Until more experimental data accumulates, further computational studies will remain difficult to validate. The methods we applied and benchmarked are suitable for re-use in much larger datasets. In the end, our most important message to medicinal chemists is that inexpensive *in silico* methods – if done at a sufficiently large scale – can point to the branching points where a large family based on a core scaffold segregates into smaller sub-series. This in turn enables limiting SAR models to compounds with common binding modes and to avoid bias from heterogenous members of the scaffold.

## Concluding Remarks

Pyrazoloquinolinones represent an old and tried chemotype with many desired properties, and have recently been associated with the exciting potential for efficacy-selective targeting of specific α or β isoforms. Progress is slowed by lacking experimental data on binding site usage, binding modes and the inherent promiscuity of many PQs. R′3-substituted PQs emerge as a particularly promising pattern for improved efficacy selectivity. We demonstrated that exhaustive exploration of posing space and pharmacophore matching of ligands to experimental structures can deliver structural hypotheses that correlate with experimental evidence, strongly suggesting that PQs interact with their binding sites with a diversity of binding modes. Switches in binding modes of similar compounds can be conceptualized as branching points in either the structure activity landscape in ligand-based SAR methods, or compounds branching out in the compound embedded space of a chemotype in a given pocket.

## Data Availability Statement

The datasets generated and analyzed for this study and not included in the article are available upon request to the authors. The R implementation of modified PHATE is available at https://github.com/MErnstLab/aPHATE for academic use.

## Author Contributions

JF and ME participated in the research design. JF, FK, and BS conducted the experiments. JF, FK, and ME wrote or contributed to writing of the manuscript. All authors performed the data analysis, contributed to the article, and approved the submitted version.

## Conflict of Interest

The authors declare that the research was conducted in the absence of any commercial or financial relationships that could be construed as a potential conflict of interest.
