# Supplementary material for: Structure-Guided Computational Methods Predict Multiple Distinct Binding Modes for Pyrazoloquinolinones in GABAA Receptors
Source: Front Neurosci. 2021 Jan 15;14:611953. doi: 10.3389/fnins.2020.611953 (PMC7844064; doi:10.3389/fnins.2020.611953)
Supplement: Supplementary file 1 [file Data_Sheet_1.pdf]

Supplementary Material

Supplementary Table 1 (provided as separate Excel file). Efficacy data for PQs extracted from the selected papers.

	**Screen 1.1**						**Screen 1.2**					
	**LigandScout [Pharmacophore-Fit score]**						**MOE [RMSD]**					
**Settings/ Ligand**	**6hup/ECD α+/γ- Diazepam**	**6huo/ECD α+/γ- Alprazolam**	**6huk/ECD ß+/α- Bicuculline methochloride**	**6D6T/ECD α+/γ- Flumazenil**	**6d6u/ECD α+/γ- Flumazenil**	**6hup/TMD ß+/α- Diazepam**	**6hup/ECD α+/γ- Diazepam**	**6huo/ECD α+/γ- Alprazolam**	**6huk/ECD ß+/α- Bicuculline methochloride**	**6D6T/ECD α+/γ- Flumazenil**	**6d6u/ECD α+/γ- Flumazenil**	**6hup/TMD ß+/α- Diazepam**
Identified features	5	8	3	6	5	3	14	18	21	18	16	14
Omitted features	3	5	1	3	3	2	8	12	14	12	10	8
Exclusion spheres	Default	Default	Off	Default	Default	Default	None	None	None	None	None	None
Ligand shape radius	Not possible in LigandScout						None	None	None	None	None	None
Flumazenil		35.61		68.75	36.11		0.2886	0.0301		0	0	0.3104
Alprazolam	46.35	88.31			35.11	36.22	0.064	0		0.0522	0.0582	0.0668
Diazepam	58.71	44.98				38.78	0	0.0634		0.3008	0.3208	0
Bicuculine			37.51					0.3989	0			
**Pyrazoloquinolinones results**												
CGS 8216					37.12							
CGS 9895					36.38					0.4086		
CGS 9896		35.41			36.37					0.408		
DCBS 32		35.63			35.05							
DCBS 76	37.55								0.1535	0.3921		
DCBS 96									0.1517	0.39		
DCBS 120												
DCBS 142					35.27		0.3461	0.379				0.3416
DCBS 146			35.06							0.316		
DCBS 152A			36.31							0.3495	0.2246	
LAU 156					36.75					0.3914		
LAU 157										0.3926		
LAU 159			36.92							0.3255		
LAU 161										0.3917		
LAU 162										0.2509	0.2217	
LAU 163												
LAU 176									0.1534	0.3516		
LAU 177									0.1522	0.3926		
LAU 206										0.3918		
LAU 462										0.3467		
PB-XHe												
PWZ-007A									0.1541			
PWZ-009A1												
PZ-II-028										0.3448		
PZ-II-029					36.34					0.3438		
XHe-II-006										0.3936		
XHe-II-17										0.3935		
XHe-II-087c												
XHe-III-006c											0.4795	
XHe-III-24												
XHe-III-063					47.14							
	**External control compounds**											
Etomidate	45.75	46.58		35.13		36.18				0.33	0.362	
Mefenamic acid	47.24	46.14		35.76	36.36	38.06						
Midazolam	48.82	78.26				38.78	0.0943	0.0265		0.0401	0.0715	0.1169

Valerenic acid	36.4	47.01		36.53		37.37				0.234		
Loreclezole												
Zaleplon												
Zolpidem												
Zopiclone												
Known high BZ affinity	1/13	1/13	-	0/13	6/13	-	0/13	0/13	-	8/13	1/13	-
Known low BZ affinity	0/2	0/2	-	0/2	0/2	-	0/2	0/2	-	1/2	0/2	-

Supplementary Table 2. Results of the pharmacophore screening with the default settings. The gray cells identify the settings that were used. The rows provide the ligands, where the first ligands are those on which the pharmacophore was based, which were also cross-screened. Bicuculline was the internal negative control. Screen results for LigandScout are the pharmacophore-fit scores, for MOE the RMSD values. The relevant cells for high benzodiazepine affinity ligands are marked in green, and yellow for low benzodiazepine affinity ligands. The relevant cells of control ligands for the transmembrane domain site (6HUP diazepam chain B associated) are colored in blue for the known binders and red for the non-binders.

	Screen 2.1	Screen 2.2	Screen 2.3	Screen 2.4
**Settings/Ligands**	**6hup/TMD ß+/α- Diazepam**			
Identified features	14	14	14	14
Omitted features	10	10	10	10
Exclusion spheres radius	None	None	None	None
Ligand shape radius	2	2.5	3	3.5
**Pyrazoloquinolinones results**				
CGS 8216	0.4161	0.285	0.262	0.1529
CGS 9895		0.2848	0.2848	0.1983
CGS 9896		0.2848	0.2848	0.1527
DCBS 32			0.2783	0.1458
DCBS 76			0.3431	0.1952
DCBS 96			0.3421	0.1957
DCBS 120		0.4187	0.2049	0.197
DCBS 142		0.4239	0.1246	0.1108
DCBS 146			0.1968	0.1964
DCBS 152A			0.1991	0.1955
LAU 156			0.1993	0.1953
LAU 157			0.2134	0.1927
LAU 159		0.4159	0.2016	0.198
LAU 161			0.2058	0.1942
LAU 162				
LAU 163		0.4288	0.2008	0.1955
LAU 176			0.3404	0.1932
LAU 177			0.3384	0.1932
LAU 206			0.2094	0.1949
LAU 462				
PB-XHe				0.3103
PWZ-007A			0.342	0.1952
PWZ-009A1			0.3015	0.2487
PZ-II-028			0.2131	0.195
PZ-II-029				
XHe-II-006				
XHe-II-17				
XHe-II-087c				0.3014
XHe-III-006c				0.312
XHe-III-24				0.2685
XHe-III-063		0.1967	0.1967	0.1967
Etomidate		0.1696	0.1696	0.1696
Mefenamic acid		0.0593	0.0472	0.0472
Midazolam	0.0583	0.0583	0.0583	0.0583
Valerenic acid	0.3328	0.3328	0.3328	0.3328
Loreclezole		0.1964	0.0921	0.077
Zaleplon			0.4468	0.4468
Zolpidem				
Zopiclone				
Nr of PQ Results	1	8	22	26

Supplementary Table 3. Pharmacophore screening into 6HUP TMD β+/α- diazepam-bound pocket (chain B associated entry) with MOE. The screens were performed by varying ligand shape parameter. The cells of control ligands are colored blue for the known binders and red for the non-binders. The used settings are reported at the top of the table (grey cells).

	Screen 2.5	Screen 2.6	Screen 2.7	Screen 2.8
**Settings/Ligands**	**6hup/TMD ß+/α-**			
Identified features	14	14	14	14
Omitted features	10	10	10	11
Exclusion spheres	1.8	1.8	1.8	1.8
Ligand shape	2.5	3	3.5	3.5
**Pyrazoloquinolinones results**				
CGS 8216	0.408	0.408	0.408	0.0709
CGS 9895			0.3742	0.2703
CGS 9896	0.3766	0.3766	0.3766	0.1602
DCBS 32				0.1465
DCBS 76				0.2783
DCBS 96				0.2792
DCBS 120		0.2422	0.2422	0.1082
DCBS 142		0.2373	0.2373	0.1048
DCBS 146			0.327	0.2491
DCBS 152A			0.3246	0.248
LAU 156			0.3702	0.2402
LAU 157			0.386	0.2502
LAU 159		0.2388	0.2388	0.1087
LAU 161			0.3755	0.2449
LAU 162				
LAU 163			0.3445	0.1211
LAU 176				
LAU 177				
LAU 206		0.2471	0.2471	0.2448
LAU 462				
PB-XHe			0.3829	0.2476
PWZ-007A				0.1199
PWZ-009A1			0.327	0.1557
PZ-II-028		0.2505	0.2505	0.2462
PZ-II-029				
XHe-II-006				
XHe-II-17				
XHe-II-087c			0.3078	0.1773
XHe-III-006c			0.312	0.1789
XHe-III-24				0.3316
XHe-III-063			0.3831	0.2454
Etomidate				0.0578
Mefenamic acid	0.0769	0.0769	0.0769	0.0345
Midazolam	0.0583	0.0583	0.0583	0.0247
Valerenic acid				0.2516
Loreclezole	0.1964	0.1964	0.1964	0.0418
Zaleplon				0.3126
Zolpidem				0.2674
Zopiclone				
Nr of PQ Results	2	7	19	24

Supplementary Table 4. Pharmacophore screening into 6HUP TMD β+/α- diazepam bound site (chain B associated entry) with different ligand shapes and exclusion spheres. The cells of control ligands are colored blue for the known binders and red for the non-binders. The used settings are reported at the top of the table (grey cells).

Settings/Ligand	Screen 3.1<br>6d6t/ECD α+/γ- Flumazenil	6d6u/ECD α+/γ- Flumazenil	Screen 3.2<br>6hup/TMD ß+/α- Diazepam
Identified features	18	16	14
Omitted features	13	11	10
Exclusion spheres	1.8	1.8	None
Ligand shape	None	None	2.5
Flumazenil	0	0	0.1449
Alprazolam	0.0142	0.0496	0.0064
Diazepam	0.1297		0.0022
**Pyrazoloquinolinones results**			
CGS 8216	0.3337	0.3477	0.285
CGS 9895			0.2848
CGS 9896	0.4206	0.4367	0.2848
DCBS 32	0.3547		
DCBS 76	0.2933	0.3879	
DCBS 96	0.2908	0.3898	
DCBS 120	0.3851	0.3685	0.4187
DCBS 142	0.3835	0.3662	0.4239
DCBS 146	0.392	0.3712	
DCBS 152A	0.3628	0.3698	
LAU 156	0.2935	0.3879	
LAU 157			
LAU 159	0.3807	0.3677	0.4159
LAU 161	0.293		
LAU 162			
LAU 163	0.333	0.3537	0.4288
LAU 176	0.3972		
LAU 177	0.2934		
LAU 206	0.2904	0.3895	
LAU 462			
PB-XHe	0.2914		
PWZ-007A	0.3335	0.3542	
PWZ-009A1	0.3329	0.3674	
PZ-II-028	0.3874		
PZ-II-029	0.388	0.5197	
XHe-II-006	0.292	0.3909	
XHe-II-17	0.291		
XHe-II-087c			
XHe-III-006c	0.2925		
XHe-III-24	0.3298		
XHe-III-063	0.2916	0.3916	0.1967
Etomidate	0.3095		0.1696
Mefenamic acid	0.2407	0.2776	0.0593
Midazolam	0.0116	0.0533	0.0583
Valerenic acid			0.3328
Loreclezole			0.1964
Zaleplon			
Zolpidem			
Zopiclone			
Known high BZ affinity	12/13	10/13	-
Known low BZ affinity	0/2	0/2	-
Nr of PQ Results	26	17	8

Supplementary Table 5. Results of the pharmacophore screening with the optimized settings. The relevant cells for high benzodiazepine affinity ligands are marked in green, and yellow for low benzodiazepine affinity ligands. The relevant cells of control ligands for the 6HUP TMD β+/α-diazepam bound site (chain B associated entry) are colored in blue for the known binders and red for the non-binders. Number of matches in each run are at the bottom of the table; used settings are provided at the top of the table (grey cells).

	Screen 4.1	
Settings/Ligand	6x3v/TMD ß+/α- (Etomidate)	
Identified features	12	12
Omitted features	8	8
Exclusion spheres	None	None
Ligand shape	2.5	3
Flumazenil	0.1473	0.024
Alprazolam		0.0097
Diazepam	0.2163	0.206
**Pyrazoloquinolinones results**		
CGS 8216	0.3396	0.242
CGS 9895		
CGS 9896	0.289	0.289
DCBS 32	0.3439	0.3439
DCBS 76	0.3138	0.3138
DCBS 96	0.3102	0.3102
DCBS 120	0.3642	0.3642
DCBS 142	0.3653	0.3653
DCBS 146		
DCBS 152A	0.419	0.419
LAU 156	0.3139	0.3139
LAU 157		
LAU 159		
LAU 161		
LAU 162		
LAU 163	0.3751	0.2695
LAU 176		
LAU 177		
LAU 206	0.3089	0.3089
LAU 462		
PB-XHe		0.3115
PWZ-007A	0.3752	0.2695
PWZ-009A1		0.372
PZ-II-028		
PZ-II-029		
XHe-II-006		0.3113
XHe-II-17		
XHe-II-087c		
XHe-III-006c		0.3107
XHe-III-24	0.3714	0.3714
XHe-III-063		
Etomidate	0.0239	0.0239
Mefenamic acid	0.2266	0.1086
Midazolam		0.021
Valerenic acid		0.2407
Loreclezole	0.4534	0.3582
Zaleplon		
Zolpidem		
Zopiclone		
Nr of PQ Results	13	17

Supplementary Table 6. Pharmacophore screening into 6X3V TMD β+/α- etomidate-bound site (chain A associated entry) with MOE with varying ligand shape settings. The cells of control ligands are colored blue for the known binders and red for the non-binders. The used settings are reported at the top of the table (grey cells).

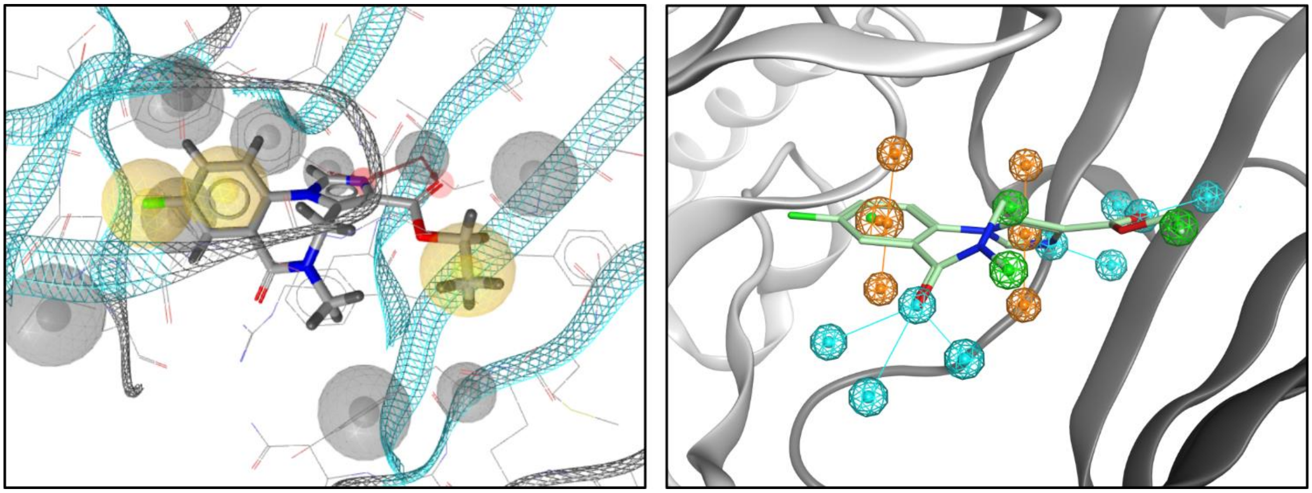

Supplementary Figure 1. Representative examples for the pharmacophore features generation by LigandScout (left) and MOE (right). Left: Ligand Scout pharmacophore derived from 6D6T with default settings. The exclusion volumes (grey spheres), hydrophobic features (yellow spheres), and H-bonding acceptor features (red spheres) are displayed. Right: MOE pharmacophore derived from 6D6T with default settings. Depicted are aromatic or pi ring features (orange spheres), hydrophobic centroids (green spheres), and H-bonding acceptor features (cyan spheres).

frontiers

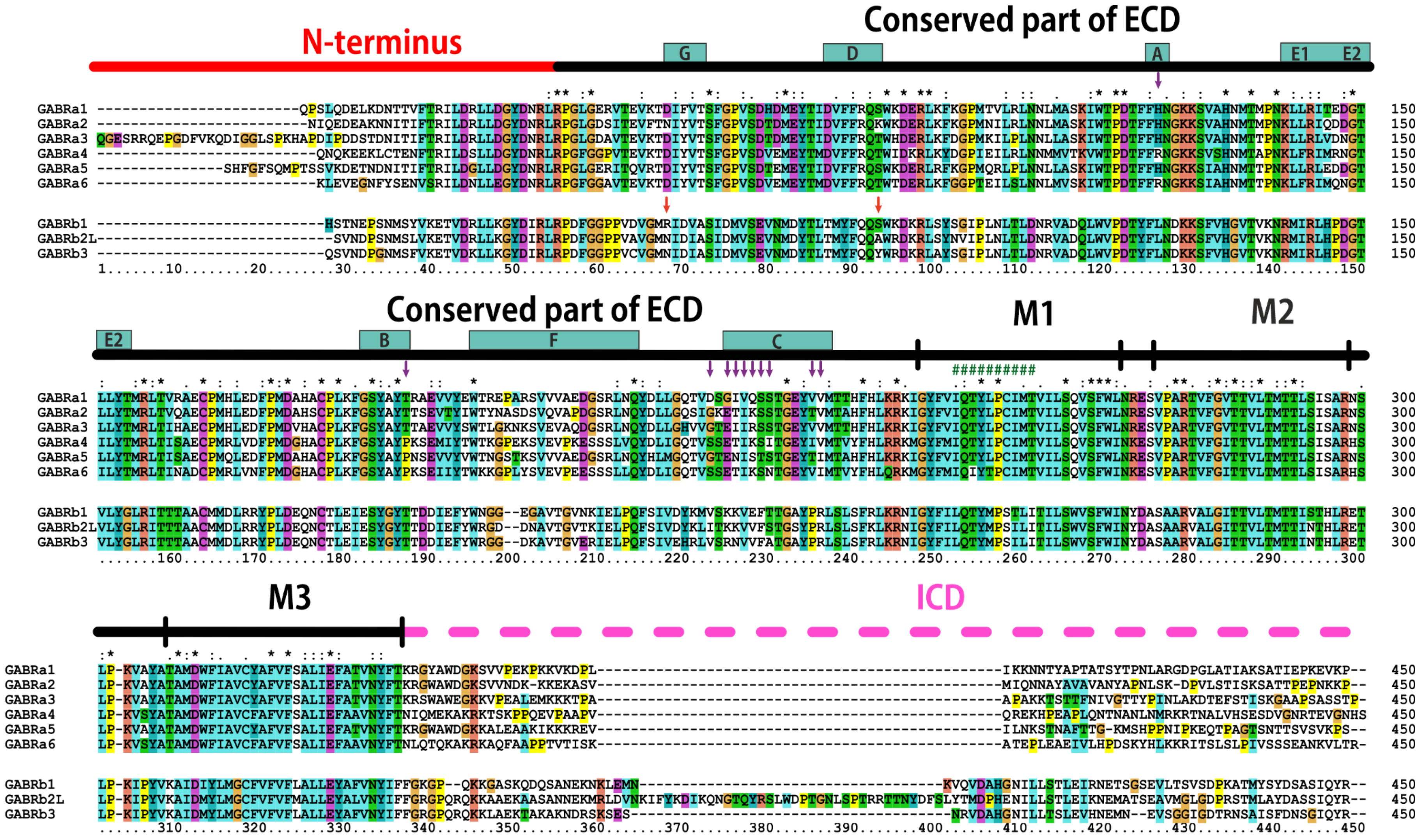
N-terminus
Conserved part of ECD
G
D
A
E1
E2
GABRa1
GABRa2
GABRa3
GABRa4
GABRa5
GABRa6
GABRb1
GABRb2L
GABRb3
Conserved part of ECD
E2
B
F
C
M1
M2
M3
ICD

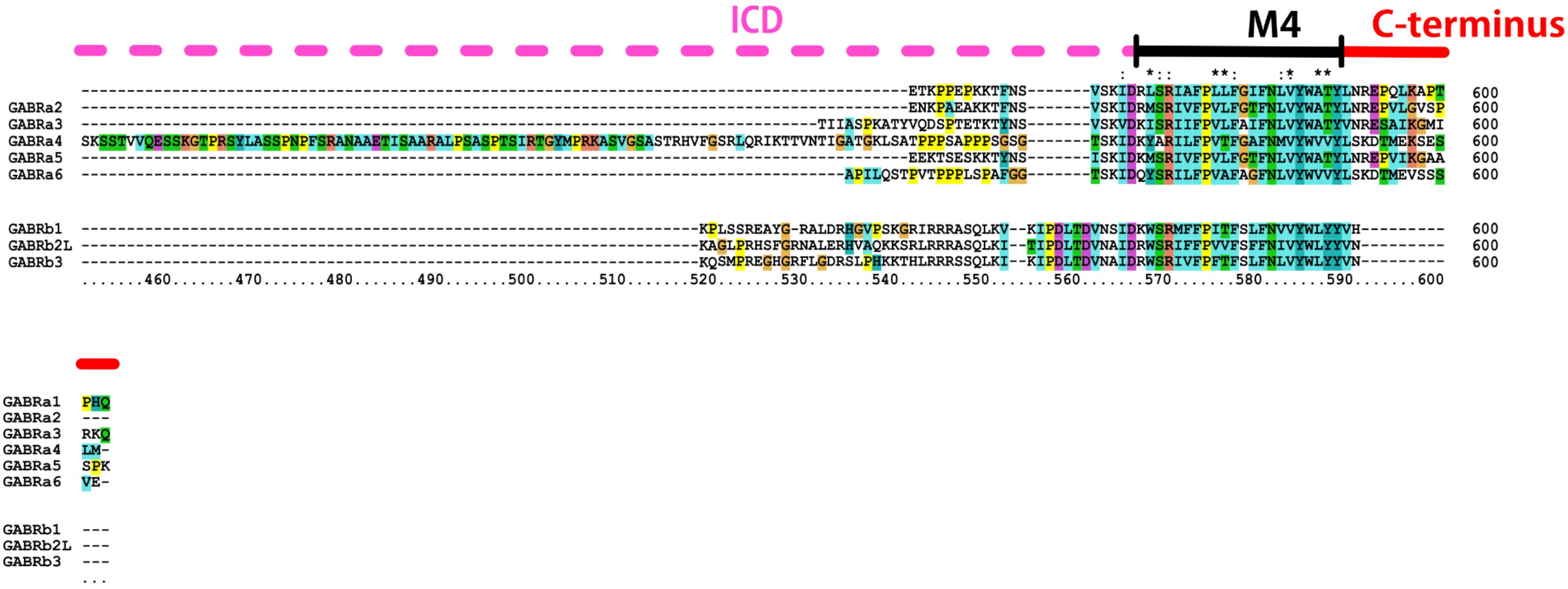

Supplementary Figure 2. Alignment of α and β subunits. Human subunits without signal peptide were used. Variable amino acids of α subunits contributing to complementary side of the TMD β+/α- pocket are marked with green #. Red arrows mark variable amino acids in β subunits contributing to ECD α+/β- pocket, while the variable amino acids contributing to this site in α subunits are marked with magenta arrows.

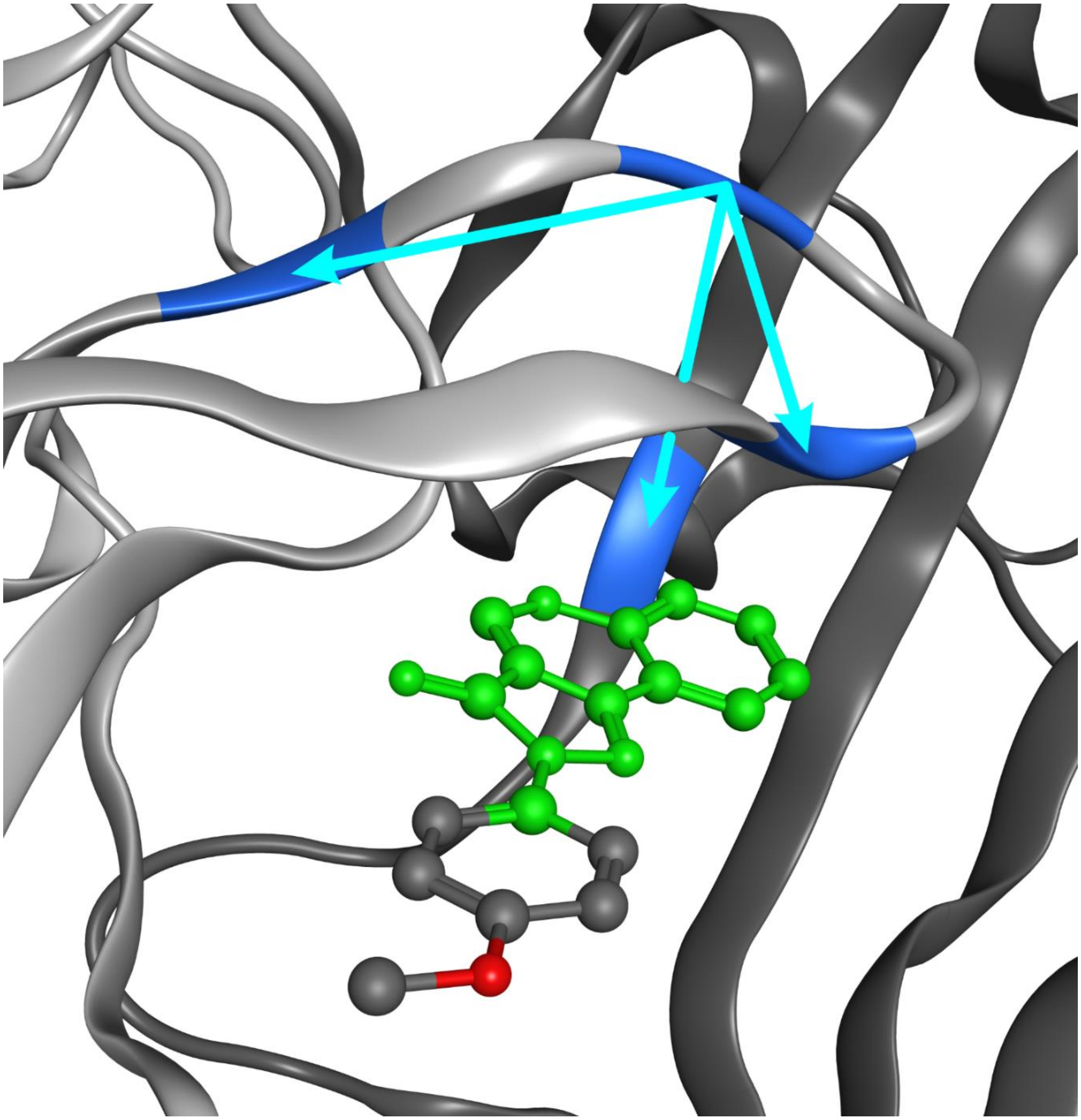

Supplementary Figure 3. Extraction of coordinates from docking poses. A representative pose of CGS 9895 in ECD α1+/β3-. Blue colored residues are used to define the coordinate system (cyan arrows), specifically alpha carbons of α1Ser205, α1Gly208, α1Tyr210, and β3Met115. The coordinates are measured for a subset of atoms common to all the docked compounds (green).

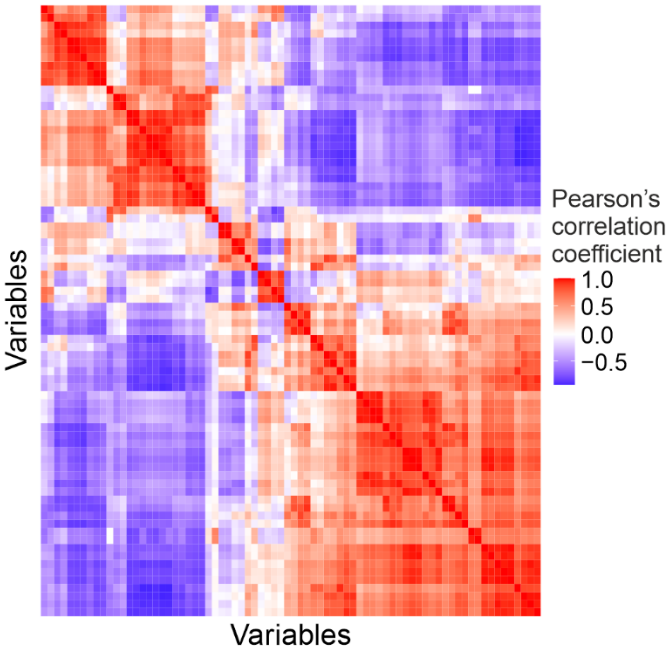

Supplementary Figure 4. Pearson's correlation between variables extracted from the computational docking results. Most variables show high level of correlation to others. 100 docking runs of PZ-II-028 in ECD α1+/β3- were performed. Spherical coordinates were extracted for selected atoms (green in Supplementary Figure 3) from all result poses (a total of 20,000 poses), yielding 4 variables per atom. Pearson's correlation coefficient was calculated for all pairs of variables

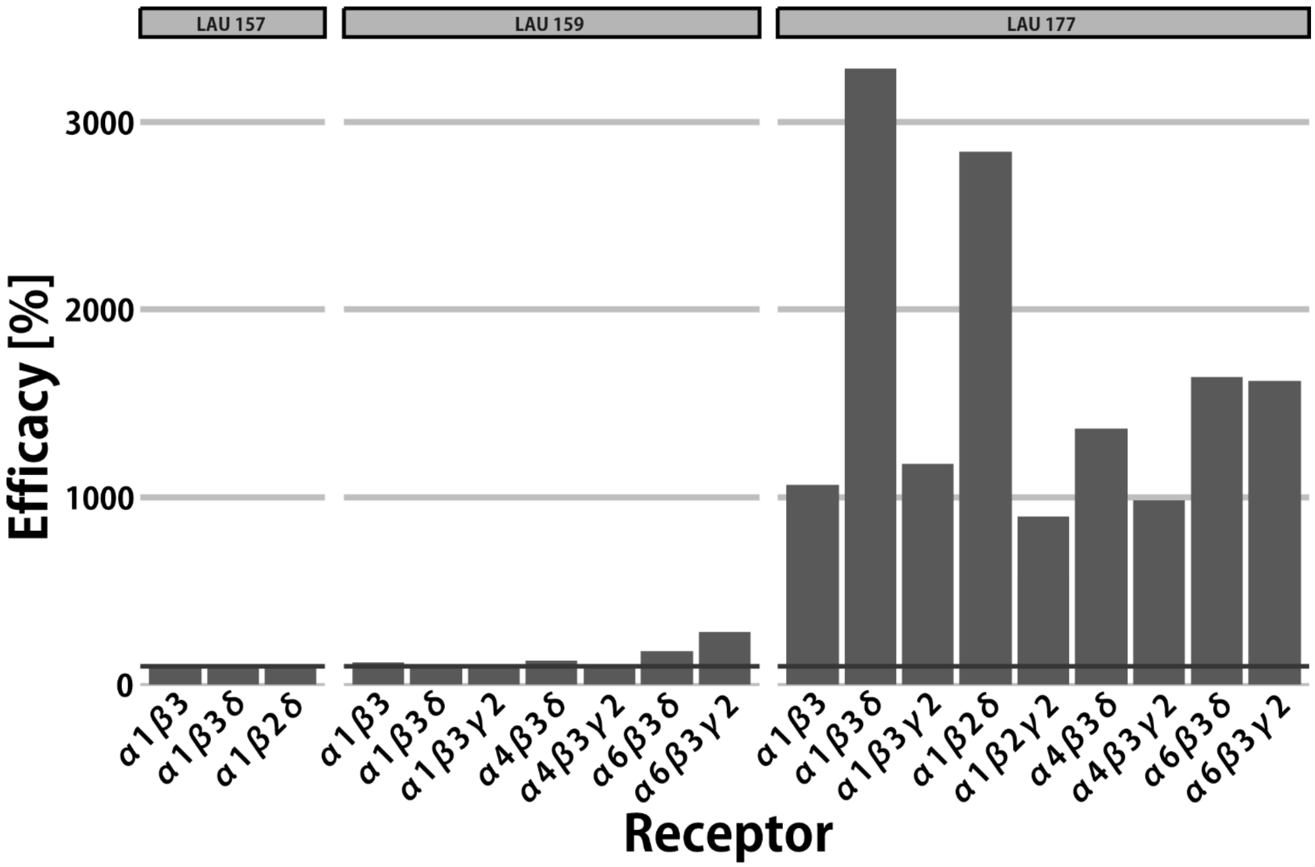

Supplementary Figure 5. Efficacies for 3 compounds with data from δ-containing receptors are available. The efficacies are depicted also for non-δ containing receptors. The compound names are stated above the graph. Grey line is placed at 100 % to delineate the point of no modulatory effect.

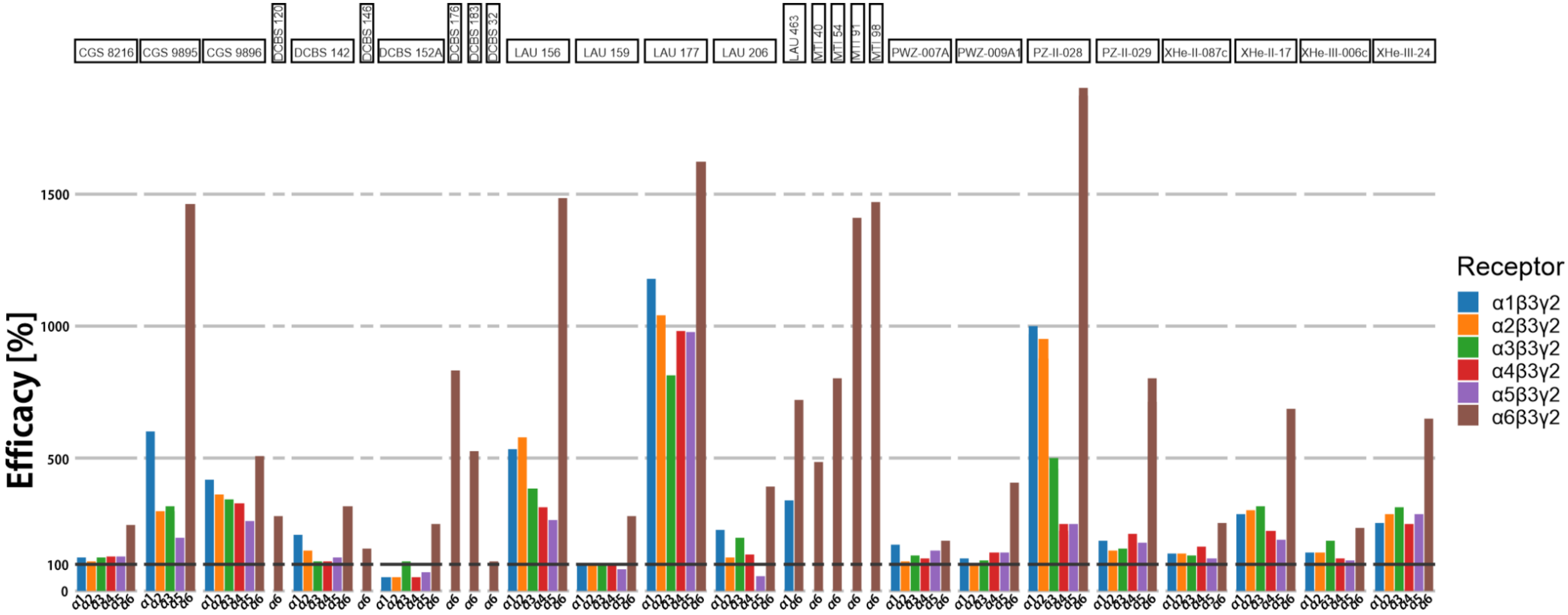

Supplementary Figure 6. Efficacies in αxβ3γ2 receptors. The compound names are stated above the graph. Note the colors represent the receptor. A black horizontal line is placed on 100 % to represent the point of no modulatory effect.

**a1S204C**		**a1Y209Q/F**		**a1V211C**		**a1S269I**	
Maldifassi 2016	CGS 9895	Maldifassi 2016	CGS 9895	Ramerstorfer 2011	CGS 9895	Maldifassi 2016	CGS 9895
Mirheydari 2014	LAU 177	Maldifassi 2016	CGS 8216	Varagic 2013a/b	PZ-II-028	Maldifassi 2016	LAU 177
		Maldifassi 2016	LAU 176	Varagic 2013a/b	XHe-II-087c		
		Maldifassi 2016	LAU 177				
**b1R41N**		**b1S290N**		**b2N265I**		**b3Q64C**	
Simeone 2017	DCBS 76	Ramerstorfer 2011	CGS 9895	Maldifassi 2016	CGS 9895	Ramerstorfer 2011	CGS 9895
Simeone 2017	DCBS 96			Maldifassi 2016	LAU 177	Varagic 2013a/b	CGS 8216
Simeone 2017	LAU 156					Varagic 2013a/b	PZ-II-028
Simeone 2017	LAU 176					Varagic 2013a/b	PZ-II-029
Simeone 2017	LAU 206					Varagic 2013a/b	XHe-II-087c
Simeone 2017	PZ-II-028					Mirheydari 2014	LAU 177
**g2M130C**		**g2S280I**					
Ramerstorfer 2011	CGS 9895	Maldifassi 2016	CGS 9895				
Varagic 2013a/b	CGS 9896	Maldifassi 2016	LAU 177				
Varagic 2013a/b	PZ-II-028						
Varagic 2013a/b	PZ-II-029						

Supplementary Table 7. List of compounds tested in mutants. Mutants are ordered in segments according to the mutated subunit with a grey row between them. The compounds are listed together with the citation.

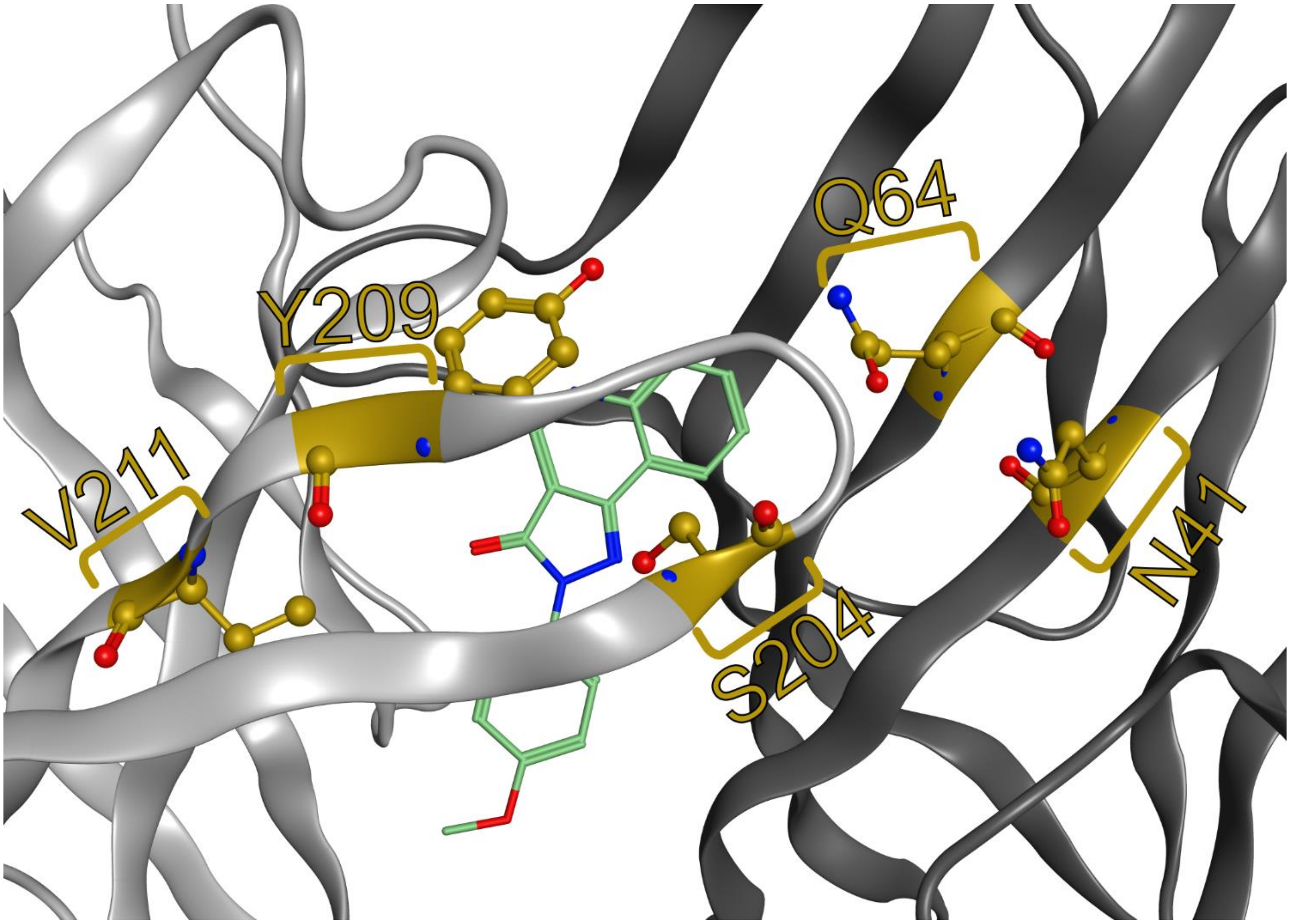

Supplementary Figure 7. ECD α1+/β3- interface; the amino acids, for which mutational data is published are displayed in yellow stick representation and marked with rat subunit numbering (as used in the majority of the papers). Note that recent structural data differs by +1 due to the difference between rat and human α sequence. To see the ligand position in relation to the mutated positions, the best scored pose of CGS 9895 in pose cluster 1.1 is displayed.

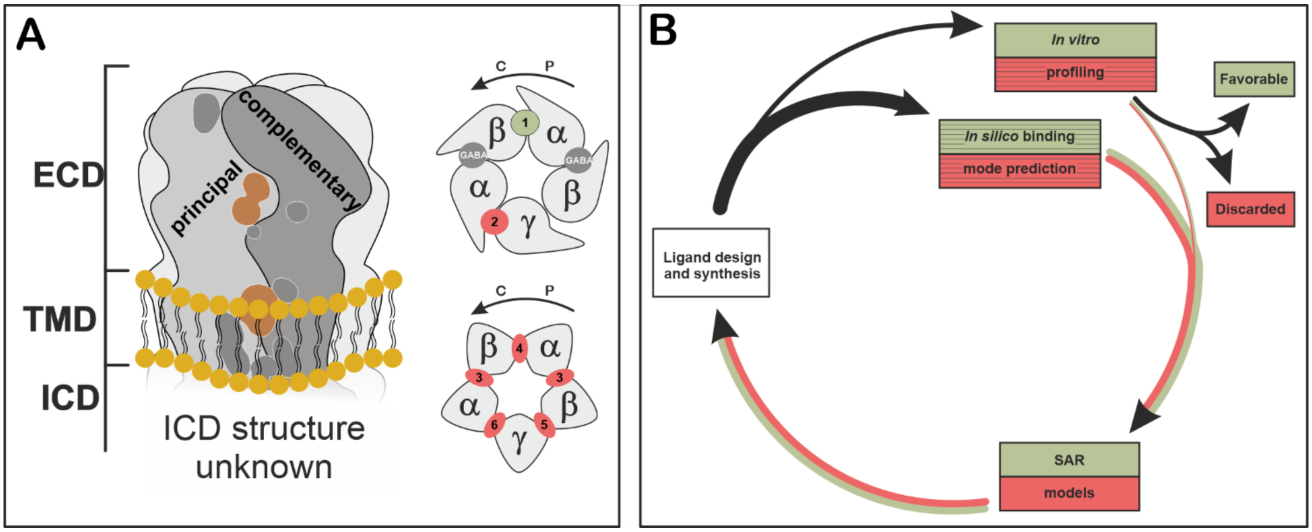

Supplementary Figure 8. (A) Schematic rendering of binding site localizations. The left panel displays a generic pentamer. ECD stands for extracellular domain, TMD for transmembrane domain and ICD for intracellular domain. The brown colored binding sites are the interface ECD- and TMD-sites which can be targeted by various compounds of the pyrazoloquinolinone class depending on the identity of the interface forming subunits. On the right-hand side are schemes of a specific pentamer consisting of α, β and γ subunits. The wanted site is depicted in green and the unwanted sites in red. (B) Schematic representation of the proposed workflow for ligand optimization. The workflow can be used for optimizing the selective targeting of ECD α+/β- pockets and accounts for additional potential interactions with all unwanted sites in the same receptor complex and comprises the following steps: (1) compound synthesis/library expansion: series with few members (e.g., R8+R'3) should be expanded, and ligands with substituents only on ring A or only on ring D should be added. (2) Full *in vitro* compound profiling: Usage of all binding sites depicted in panel A needs testing to attribute efficacy correctly to one or more sites. The desired site is represented by green processes, the unwanted sites by red ones. The usage of TMD sites is unknown for most published PQs, see Supplementary Table 7. (3) In silico binding mode prediction: Binding mode candidates per ligand for each used binding site are generated by fast methods (pharmacophore matching where possible, else computational docking). The aim of this part of workflow is not the prediction of the bound state structure by the use of more advanced methods, as this would be too time consuming. However, if used, docking runs must be large enough to adequately sample the posing space. This step assigns individual compounds to one or more candidate binding modes in each site. More compounds can be used compared to the *in vitro* profiling to assist in step 5. For ambiguously posing compounds, multiple candidates are retained for the next step. (4) Structure-activity models: The predictions from (3) and the data from (2) are used in SAR models. For ligands which share a common binding mode, the model should converge while wrongly posed ligands will be outliers. Competing SAR models can arise from several sources: Compounds which display similar activity result in "flat SAR"; prediction of multiple candidate poses leads to alternative SAR models. (5) Next generation compound synthesis: To test promising SAR models, a next iteration is made. Correctly identified features that produce unfavorable interactions should eliminate binding at the unwanted sites. If the next iteration of *in vitro* profiling confirms the SAR models, compounds with desired profiles are kept while compounds with high level of promiscuity are discarded. For compounds on branching points of the SAR or outliers in the posing space, additional analogues are needed.

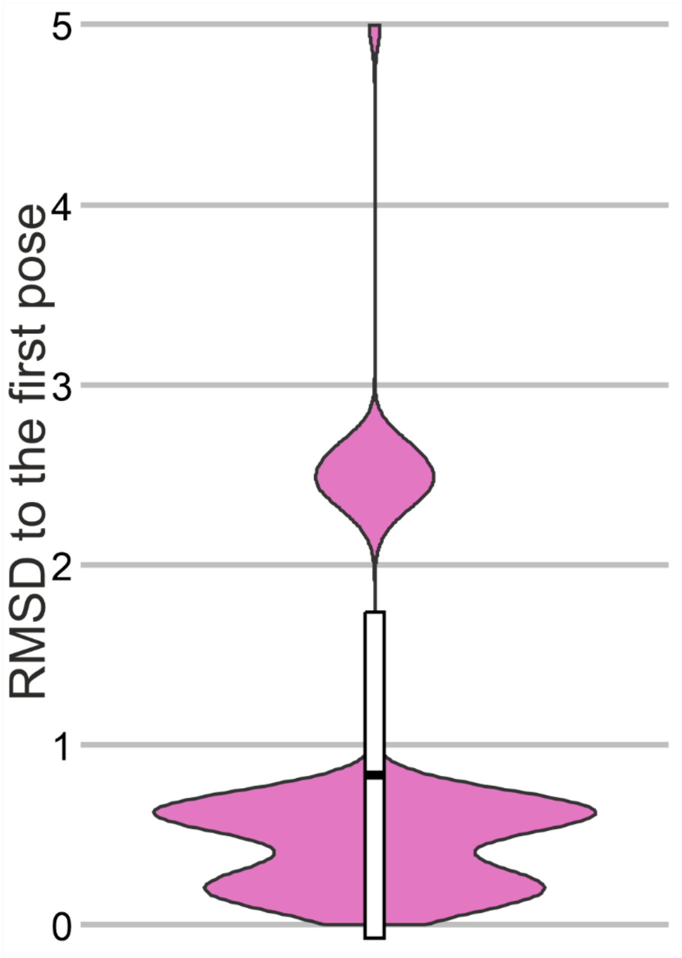

Supplementary Figure 9. RMSD comparison between top scoring poses from 100 separate docking runs. 100 docking runs of PZ-II-028 in the ECD α1+/ β3- pocket with the same configuration as described in the methods section were performed. From each run the top scored pose according to chemscore was extracted, and RMSD of the selected poses was calculated in relation to the pose from the first run. The distribution of the RMSD is plotted in pink, while the box plot depicts the average and standard deviation of RMSD. Although most poses are similar, 20 % of them fall into RMSD > 1.

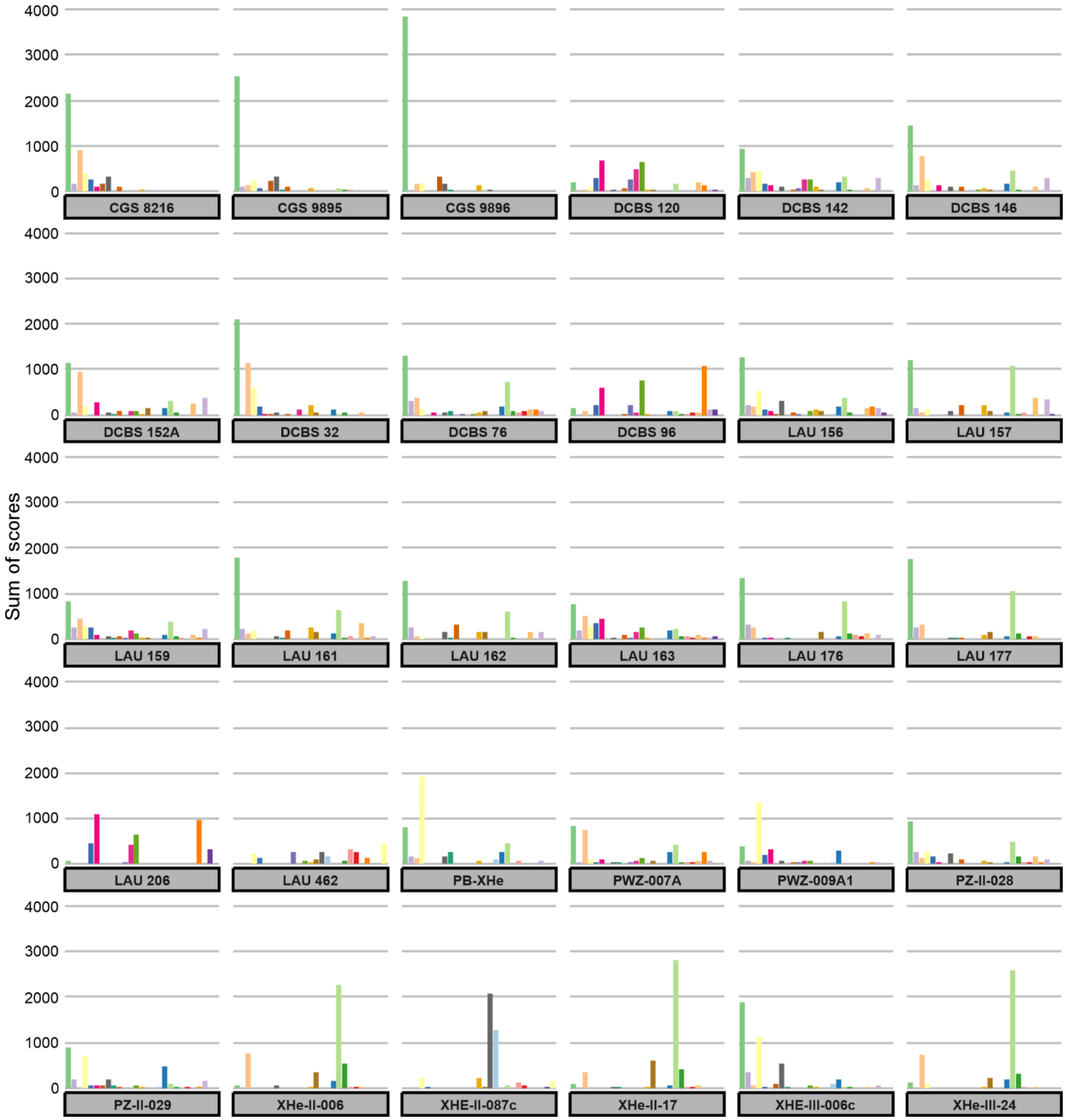

Supplementary Figure 10. Sum of score distributions for docking of compounds in ECD α1+/β3-binding site. The distributions are given for all the compounds used in docking. The compound names are stated below the individual graphs. Cluster color coding is as in Figures 7 and 8 in the manuscript.

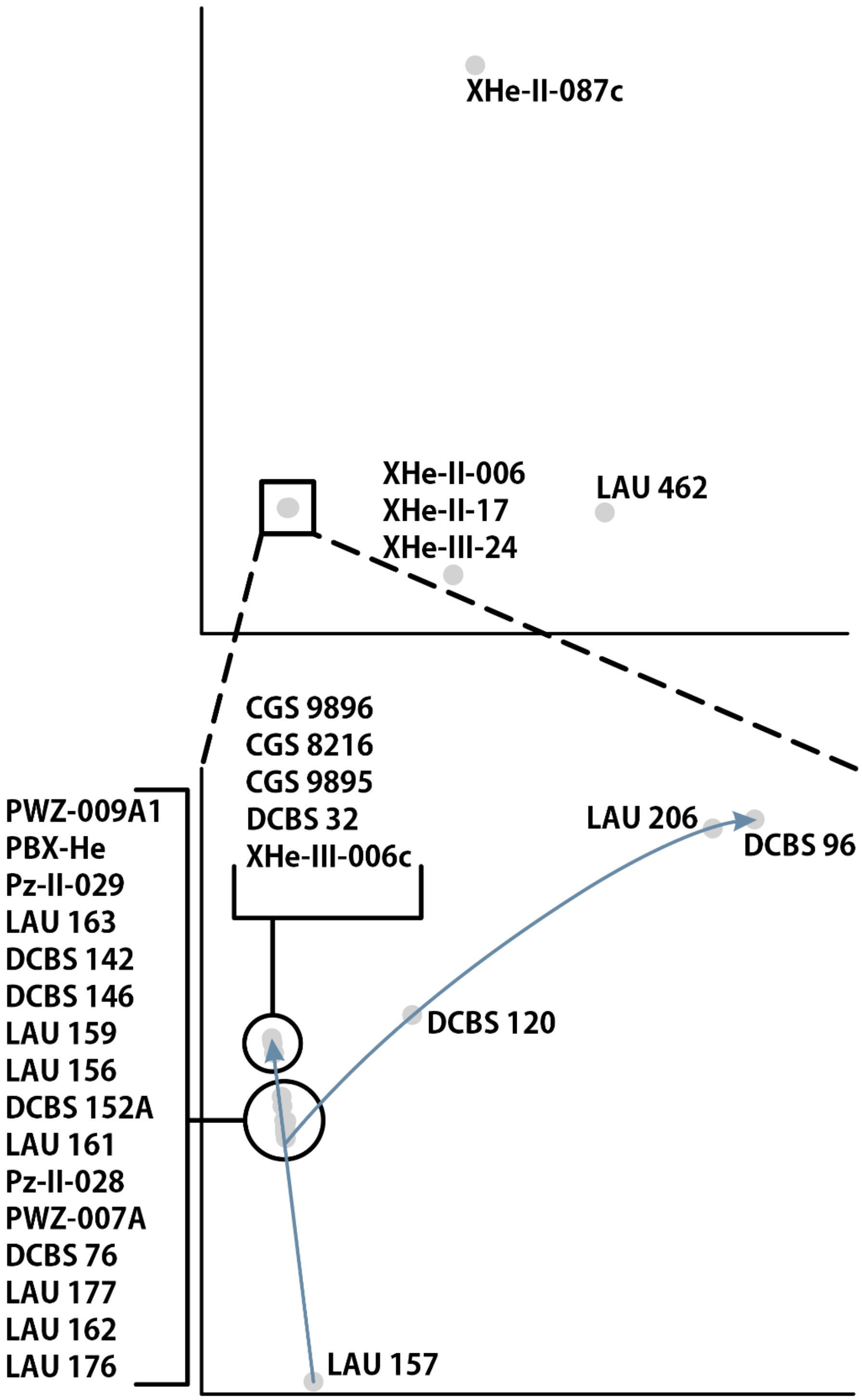

Supplementary Figure 11. Compound embedding for ECD α1+/β3- docking. All compounds are listed to help localizing them in the compound clusters. The upper panel shows the whole compound space, while the lower one is a zoomed-in view of the encircled part of the upper. The arrows show the continuous axes of changes in score distribution across the clusters.

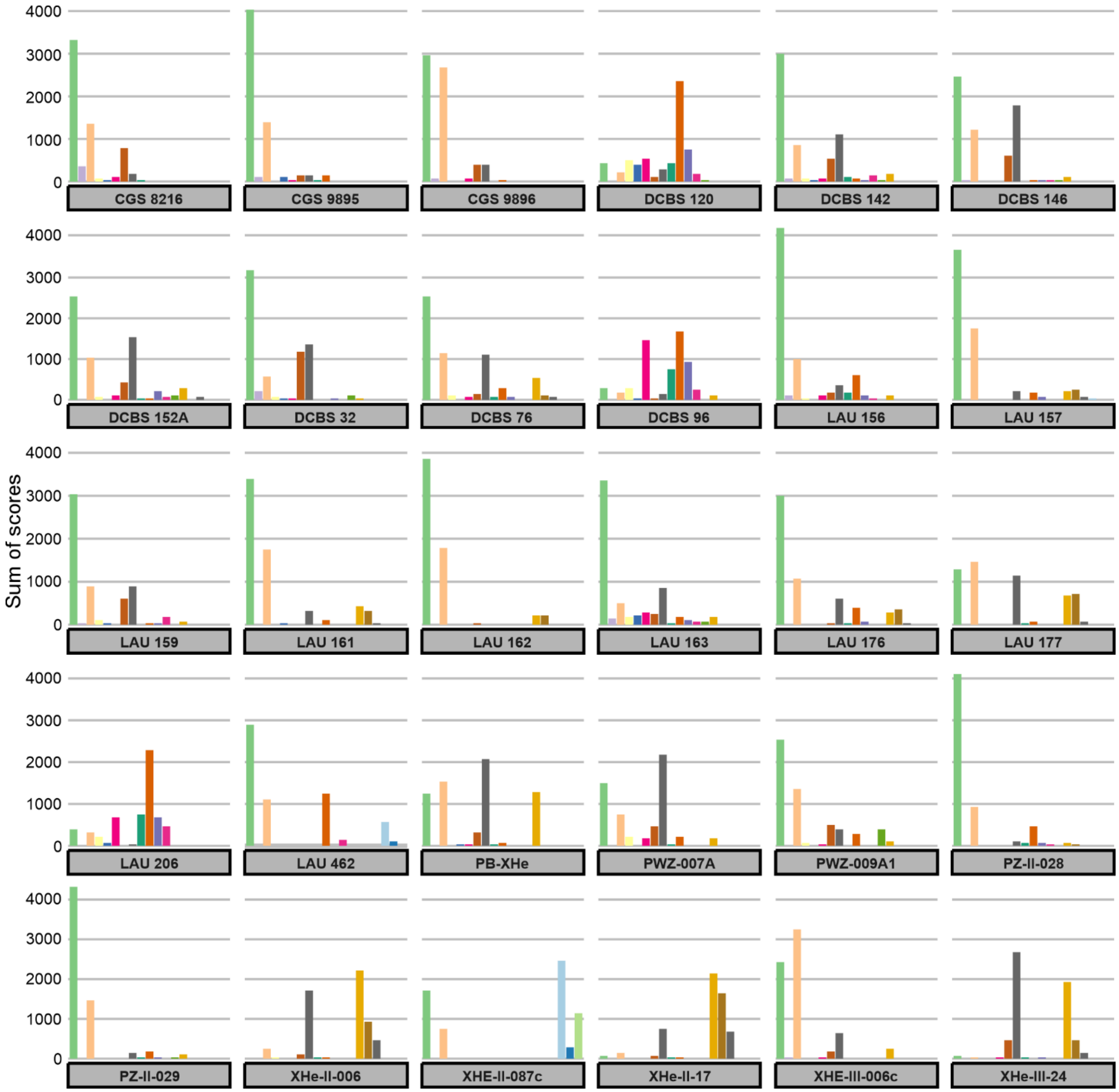

Supplementary Figure 12. Sum of score distributions for docking of compounds in ECD α6+/β3- binding site. The distributions are given for all the compounds used in docking. The compound names are stated below individual graphs. The compound names are stated below the individual graphs. Cluster color coding is as in Figures 7 and 9 in the manuscript.

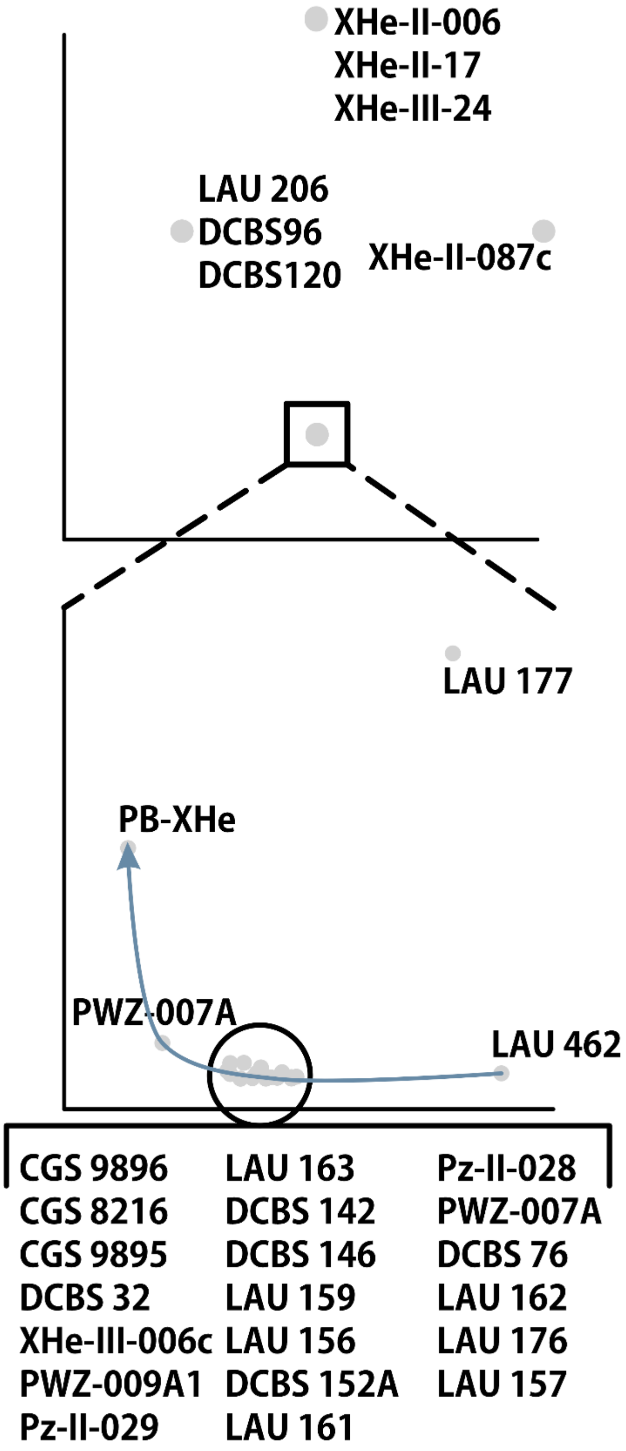

Supplementary Figure 13. Compound embedding for ECD α6+/β3- docking. All compounds are listed to help localizing them in the compound clusters. The upper panel shows the whole compound space, while the lower one is a zoomed-in view of the encircled part of the upper. The arrows show the continuous axes of changes in score distribution across the clusters.

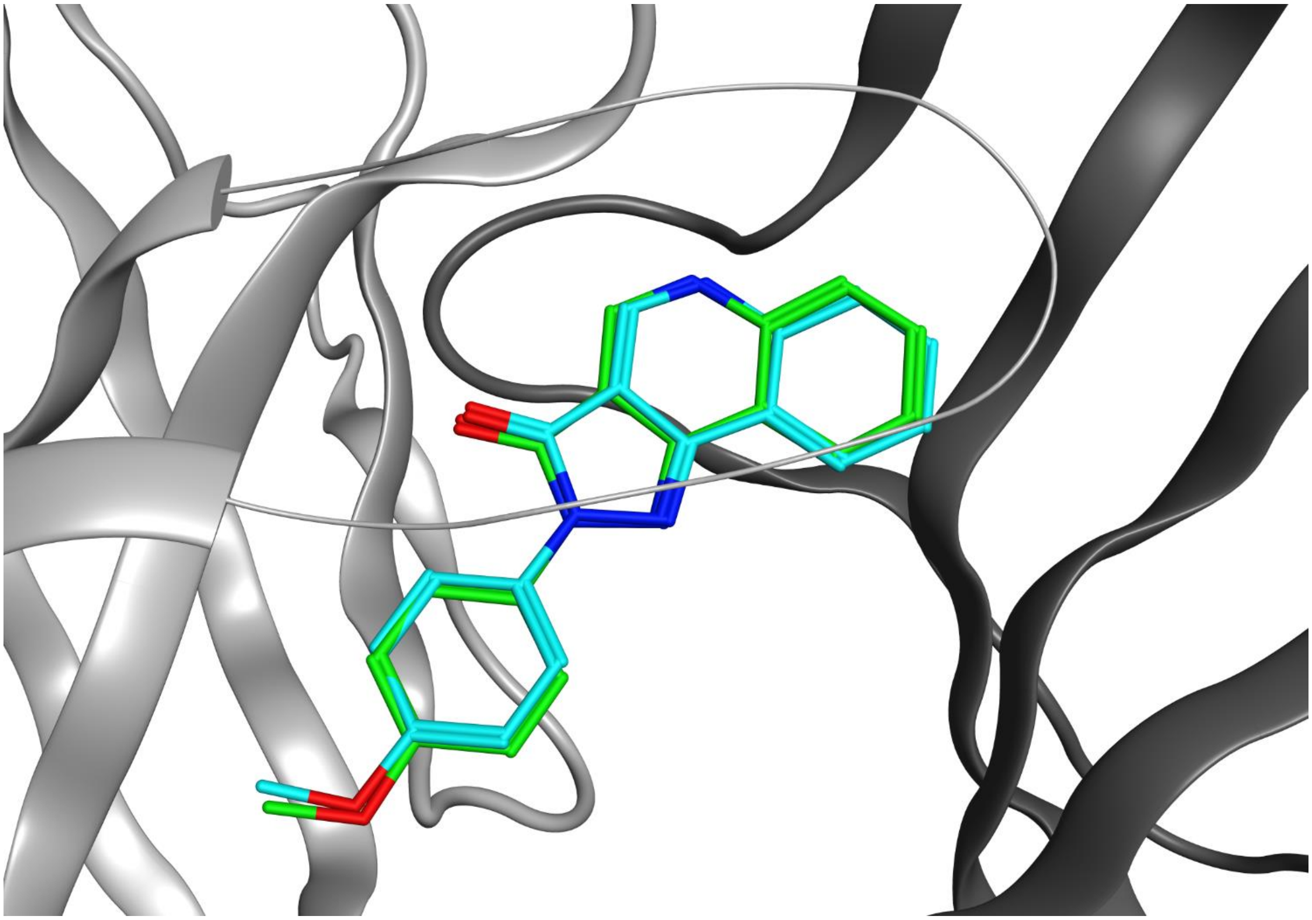

Supplementary Figure 14. Representative poses of CGS 9895 in pose cluster 1 from ECD α1+/β3- (cyan) and ECD α6+/β3- (green) docking. Both clusters have the highest accumulated score in their respective runs when pooling all the compounds together. The selected poses have the lowest RMSD when comparing pose clusters 1.1 and 1.6.

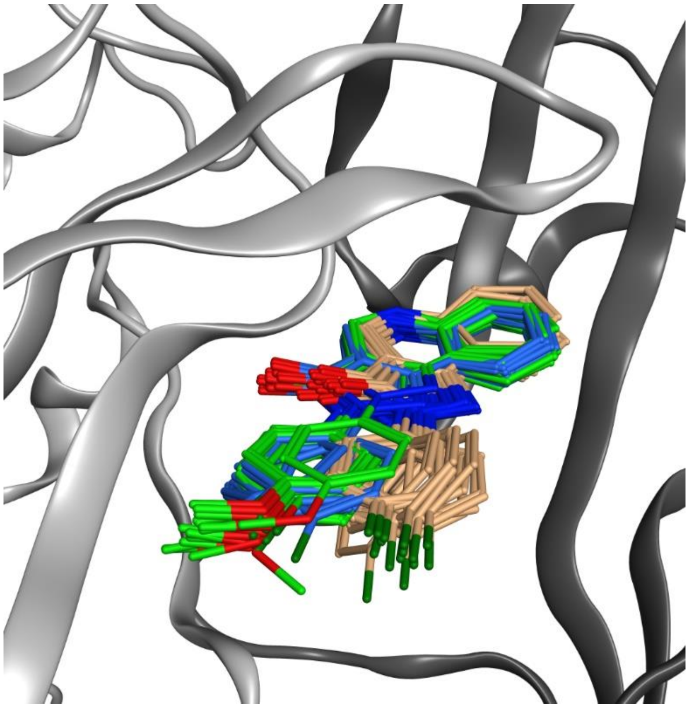

Supplementary Figure 15. Top 20 scoring poses of CGS 9895 in pose cluster 6.1 (green), and CGS 9896 in pose clusters 6.1 (blue) and 6.3 (orange) from ECD α6+/β3- docking

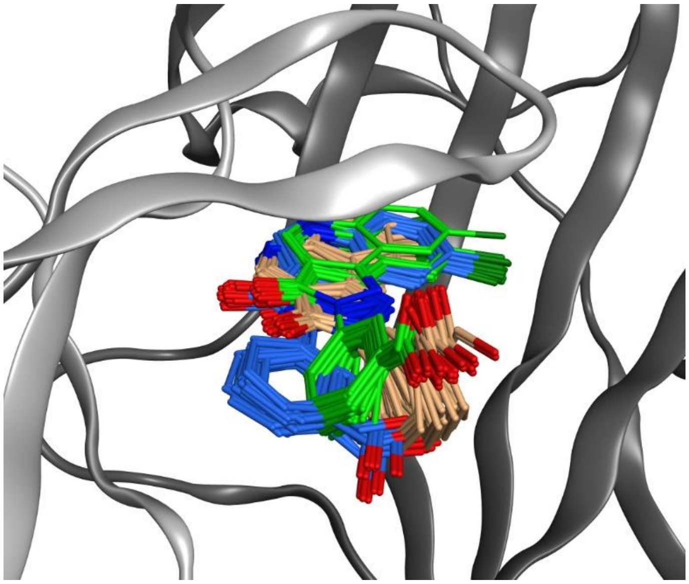

Supplementary Figure 16. Top 20 scoring poses of DCBS 152A in ECD α1+/β3- docking pose clusters 1.1 (green) and 1.3 (orange), and in ECD α6+/β3- docking pose cluster 6.1 (blue).

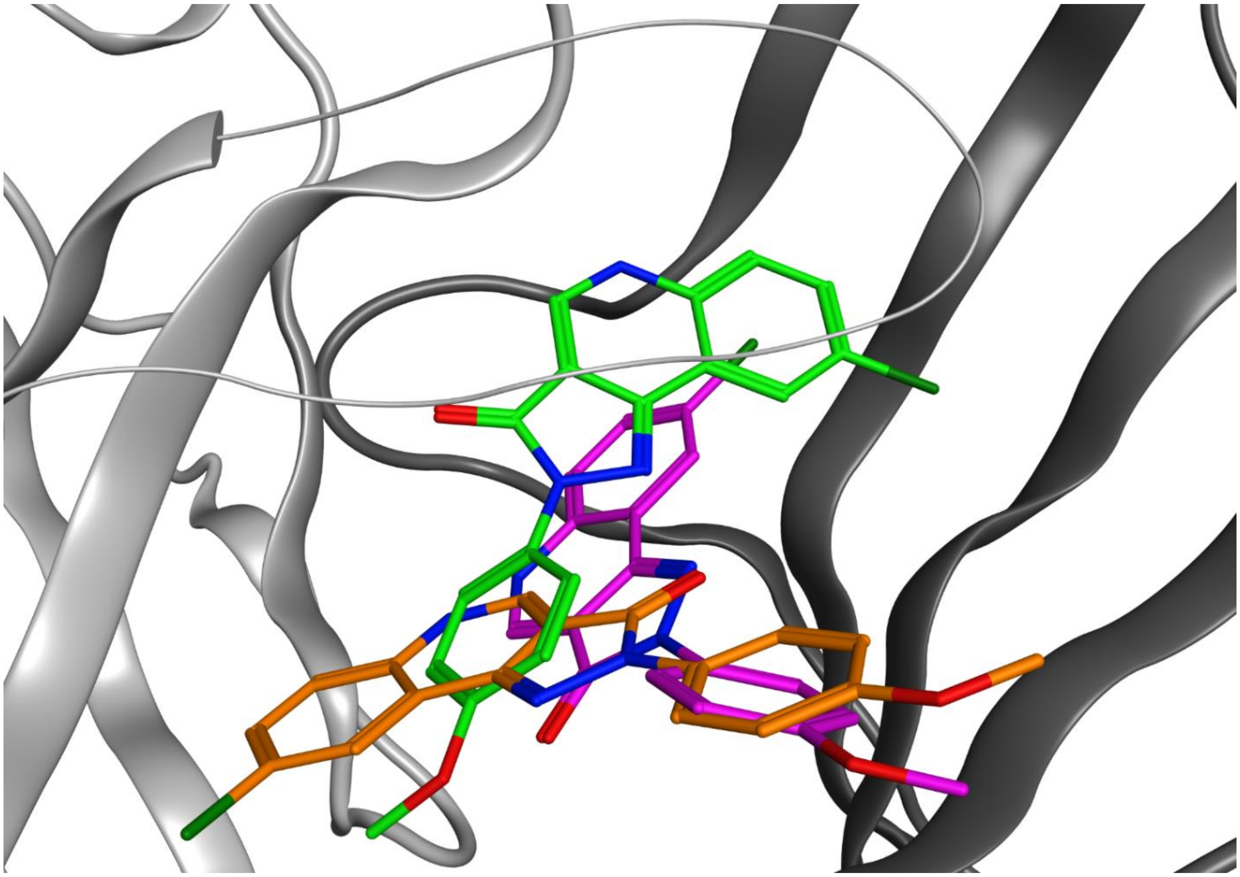

Supplementary Figure 17. Best scoring poses of PZ-II-028 in pose clusters 1.1 (green), 1.19 (magenta) and 1.23 (orange) from ECD α1+/β3- docking. In a recently published study (49) PZ-II-028 was docked, and EC50 values of a small panel of ligands were used to correlate predicted and observed potencies. The goal was to identify the "best common binding mode" for all the included compounds, resulting in the emergence of two best binding modes (BM1 and BM2). Compared to the results obtained by our analysis, the best binding mode (BM1) closely replicates pose cluster 1.23, while the second-best binding mode (BM2) could be linked to 3 pose clusters, of which cluster 1.19 contains the highest sum of score in PZ-II-028 (Supplementary Figures 10 and 17). Finding both binding modes in the posing clusters indicates that both studies sampled the posing space comprehensively. On the other hand, according to our results PZ-II-028 prefers pose cluster 1.1 over 1.19 or 1.23 (Supplementary Figure 10). Important to note is also the big difference in ligand orientation between the mentioned pose clusters (Supplementary Figure 17). Singh and Villoutreix (49) use an analysis, which tries to exploit the assumption of a common binding mode for the whole ligand class. In contrast, our analysis pipeline does not impose such a restriction on the data. Thus, the two studies in combination can be interpreted as evidence for a limited number of binding modes, which can be common to narrow sub-series of a large pool of derivatives in a SAR landscape with compound clusters of different sizes connected by branches.
